# Unraveling the evolution of photogenerated holes from charge separation to interfacial transfer in photocatalysis

**DOI:** 10.1093/nsr/nwag379

**Published:** 2026-06-18

**Authors:** Fengke Sun, Yue Zhao, Xianchang Yan, Panwang Zhou, Can Li, Rengui Li, Shengye Jin, Wenming Tian

**Affiliations:** State Key Laboratory of Chemical Reaction Dynamics, Dalian Institute of Chemical Physics, Chinese Academy of Sciences, Dalian 116023, China; University of Chinese Academy of Sciences, Beijing 100049, China; State Key Laboratory of Catalysis, Dalian National Laboratory for Clean Energy, iChEM (Collaborative Innovation Center of Chemistry for Energy Materials), Dalian Institute of Chemical Physics, Chinese Academy of Sciences, Dalian 116023, China; ZJU-Hangzhou Global Scientific and Technological Innovation Center, Zhejiang University, Hangzhou 311200, China; State Key Laboratory of Catalysis, Dalian National Laboratory for Clean Energy, iChEM (Collaborative Innovation Center of Chemistry for Energy Materials), Dalian Institute of Chemical Physics, Chinese Academy of Sciences, Dalian 116023, China; State Key Laboratory of Catalysis, Dalian National Laboratory for Clean Energy, iChEM (Collaborative Innovation Center of Chemistry for Energy Materials), Dalian Institute of Chemical Physics, Chinese Academy of Sciences, Dalian 116023, China; State Key Laboratory of Catalysis, Dalian National Laboratory for Clean Energy, iChEM (Collaborative Innovation Center of Chemistry for Energy Materials), Dalian Institute of Chemical Physics, Chinese Academy of Sciences, Dalian 116023, China; State Key Laboratory of Chemical Reaction Dynamics, Dalian Institute of Chemical Physics, Chinese Academy of Sciences, Dalian 116023, China; State Key Laboratory of Chemical Reaction Dynamics, Dalian Institute of Chemical Physics, Chinese Academy of Sciences, Dalian 116023, China

**Keywords:** carrier dynamics, transient reflection microscopy, photocatalytic mechanism, bismuth vanadate

## Abstract

A photocatalytic process involves the intricate integration of complex photophysical and photochemical events that occur at nanoscale interfaces and evolve over femtoseconds to second scales. However, directly tracking the nanoscale carrier dynamics of a single photocatalyst crystal under the reaction conditions is essential but remains challenging. Here, we employed *in situ* transient reflection microscopy to spatiotemporally resolve the evolution of photogenerated carriers in facet-engineered bismuth vanadate (BiVO_4_) photocatalyst. It is revealed that photogenerated holes undergo three sequential steps: initial ultrafast charge separation (∼5 ps), hole trapping (∼1.5 ns) and trap states‐mediated interfacial transfer to pre-adsorbed H_2_O on the surface (∼19 ps). Crucially, trap states associated with oxygen lattice sites act as nanoscale relay sites that enable rapid hole injection into pre-adsorbed H_2_O, whereas direct valence-band hole injection is negligible. These findings provide a comprehensive mechanistic picture of photogenerated carrier dynamics across the entire photocatalytic process, directly linking nanostructure to interfacial chemistry and revealing trap states as critical mediators of efficient interfacial charge transfer.

## Teaser

This work reveals the spatiotemporal evolution of photogenerated holes in bismuth vanadate under reaction conditions, highlighting defect engineering as an effective strategy to accelerate interfacial hole transfer and enhance photocatalysis.

## INTRODUCTION

Artificial photosynthesis offers a compelling strategy for converting solar energy into renewable chemical fuels [1–4]. In a typical photocatalytic reaction, the solar-to-fuel efficiency is often limited by the kinetic competition between rapid carrier recombination and slow interfacial charge transfer and catalytic reaction [5]. The pronounced mismatch between photogenerated carrier lifetime (picoseconds to nanoseconds) and the much slower catalytic reaction timescales (milliseconds to seconds) has driven extensive efforts to extend carrier lifetimes in photocatalysts [6,7]. Among the strategies developed, ultrafast facet-dependent charge separation in representative photocatalysts (e.g. Cu_2_O and BiVO_4_) stands as an effective pathway for extending carrier lifetime and thereby improving the photon utilization efficiency [8,9].

Despite the understanding of semiconductor carrier dynamics, photocatalytic reactions also involve a series of complicated photophysical and photochemical processes occurring at the nanoscale solid/liquid interface [10,11]. Conventional time-resolved techniques have limited access to these processes under reaction conditions; therefore, interfacial dynamics are generally inferred indirectly from steady-state measurements such as electrochemical methods, *in situ* infrared or Raman spectroscopy [12–14], which lack the temporal resolution to capture the mechanistic ultrafast interfacial steps. To overcome this limitation, recent work has shifted attention from bulk behavior to interface and revealed diverse pathways for interfacial charge evolution. For example, Li *et al.* revealed that trap states from surface hydroxyl groups in BiOCl enable charge separation with trapped holes persisting for tens of milliseconds and potentially supporting slow oxidation reactions [15], while Cuk *et al.* observed the ultrafast (1.3 ps) conversion of photogenerated holes into surface radicals, suggesting a rapid hole-to-reactant transfer pathway [16,17].

However, almost all of these experimental studies address only part of the photocatalysis process, and their generality across materials and reaction conditions remains untested. Consequently, there exists a gap in mechanistic understanding between semiconductor physics (generation and separation of carriers) [7,18] and surface chemistry (interfacial catalytic reactions) [19,20]. Within this gap lie a range of complex interfacial processes, including trapping, polaron formation, defect-induced carrier recombination and slow interfacial charge transfer, that remain poorly understood and can introduce significant energetic losses [10,11,21–25]. Overlooking these processes obscures the true kinetic bottlenecks, potentially misleading catalyst design. Moreover, the limited transport distance of photogenerated carriers restricts the photocatalyst particles to nano-microscale, which prevents the conventional macroscopic techniques from accessing the critical interfacial dynamics. To elucidate and overcome these bottlenecks, it is crucial to track the complete spatiotemporal evolution of photogenerated carriers with nanoscale resolution under realistic reaction conditions.

To bridge the gap, we investigate the full evolution pathway of photogenerated carriers in a facet-engineered decahedral single-crystal BiVO_4_ photocatalyst as a model system, owing to its efficient charge separation and high water-oxidation activity. Using a home-built pump-probe transient reflection microscope (TRM), we precisely mapped hole dynamics at specific facets with nanometer resolution and elucidated their evolution pathway from initial generation to participation in surface reactions. Our results identify three successive stages of hole evolution: (i) ultrafast photogenerated charge separation within 5 ps leads to hole accumulation on the {110} facets; (ii) subsequent trapping of holes into trap states on a timescale of ∼1.5 ns and (iii) a defect-mediated interfacial transfer of holes to adsorbed H_2_O molecules within 19 ps. Notably, direct valence-band hole transfer is negligible, indicating that surface defects serve as nanoscale relay sites that enable efficient photocatalytic oxidation. These findings deliver a comprehensive spatiotemporal picture of hole evolution in BiVO_4_ and point to defect engineering as a targeted strategy to accelerate interfacial charge transfer and improve photocatalytic performance.

## RESULTS AND DISCUSSION

### Steady-state evaluation of photocatalytic performance

**Figure 1.**
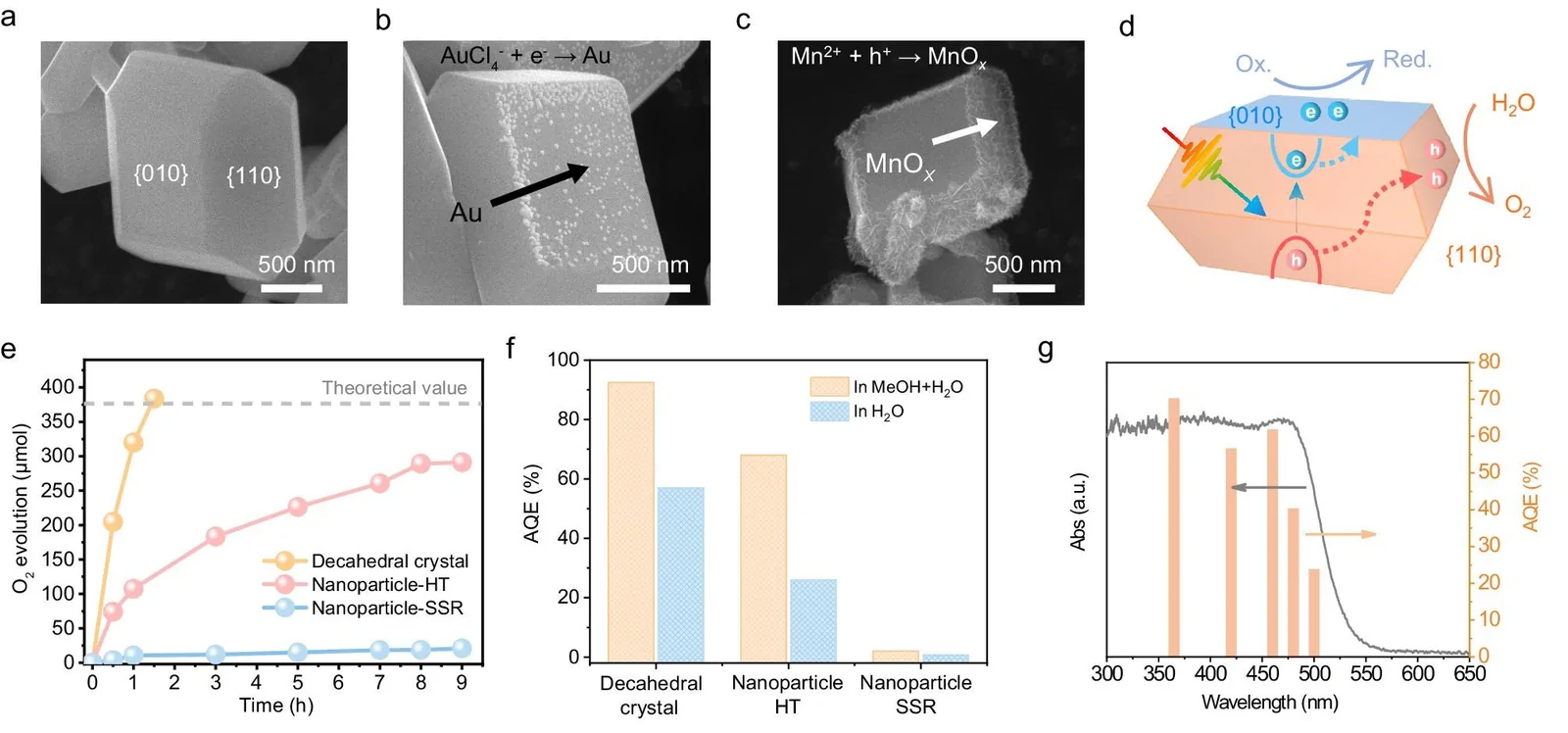
Photogenerated charge distribution and photocatalytic activities of facet-engineered BiVO_4_ crystals. (a) Scanning electron microscopy (SEM) images of decahedral BiVO_4_. (b, c) SEM images of decahedral BiVO_4_ after *in situ* (b) photoreduction deposition of Au (0.5 wt%) and (c) photooxidation deposition of MnO_x_ (1.0 wt%). Scale bars are 500 nm. (d) Schematic illustration of charge separation and the water-oxidation reaction occurring on decahedral BiVO_4_. (e) Photocatalytic water-oxidation performance for decahedral BiVO_4_ crystal and nanoparticles (Nanoparticle-HT was synthesized using the hydrothermal method and Nanoparticle-SSR was synthesized using the solid-state reaction). (f) AQE of charge separation and water oxidation for the decahedral crystal and nanoparticles. (g) AQEs of photocatalytic water oxidation for decahedral BiVO_4_ and its absorbance under different wavelengths. HT, hydrothermal; SSR: solid-state reaction.

Facet engineering-induced spatial charge separation in BiVO_4_ crystals directs surface reduction and oxidation reactions to distinct facets, rendering decahedral BiVO_4_ with predominant {010} and {110} exposure an ideal model for studying interfacial charge-transfer kinetics [26,27]. In this work, monoclinic decahedron BiVO_4_ crystals with well-defined {010} and {110} facets were synthesized (Fig. 1a and Fig. S1). *In situ* probe reactions, involving AuCl_4_^−^ photoreduction and Mn^2+^ photooxidation (Fig. 1b and c), unambiguously demonstrate that, in the as-prepared BiVO_4_, photogenerated electrons accumulate on the {010} facets to participate in the reduction reaction, while holes preferentially migrate to the {110} facets where oxidation reactions occur (Fig. 1d). Photocatalytic water-oxidation measurements (Fig. 1e) show that the decahedral BiVO_4_ exhibits markedly enhanced activity compared to BiVO_4_ nanoparticles lacking spatial charge separation (Fig. S2). Corresponding quantum efficiency tests show that the decahedral BiVO_4_ achieves ∼92% charge-separation efficiency and an apparent quantum efficiency (AQE) of ∼57% for water oxidation, significantly outperforming non-faceted samples (Fig. 1f). Notably, this high AQE is well-maintained across the absorption range (Fig. 1g). Collectively, these results establish that the spatial separation of reduction and oxidation sites on the decahedron BiVO_4_ crystal, combined with near-unity charge-separation efficiency and robust water-oxidation activity that enable most photogenerated carriers to participate in subsequent photocatalytic reactions, provides a simple yet powerful platform for elucidating the behaviors of photogenerated electrons and holes in complex photocatalytic processes.

### Spatiotemporal evolution of photogenerated holes in BiVO_4_

**Figure 2.**
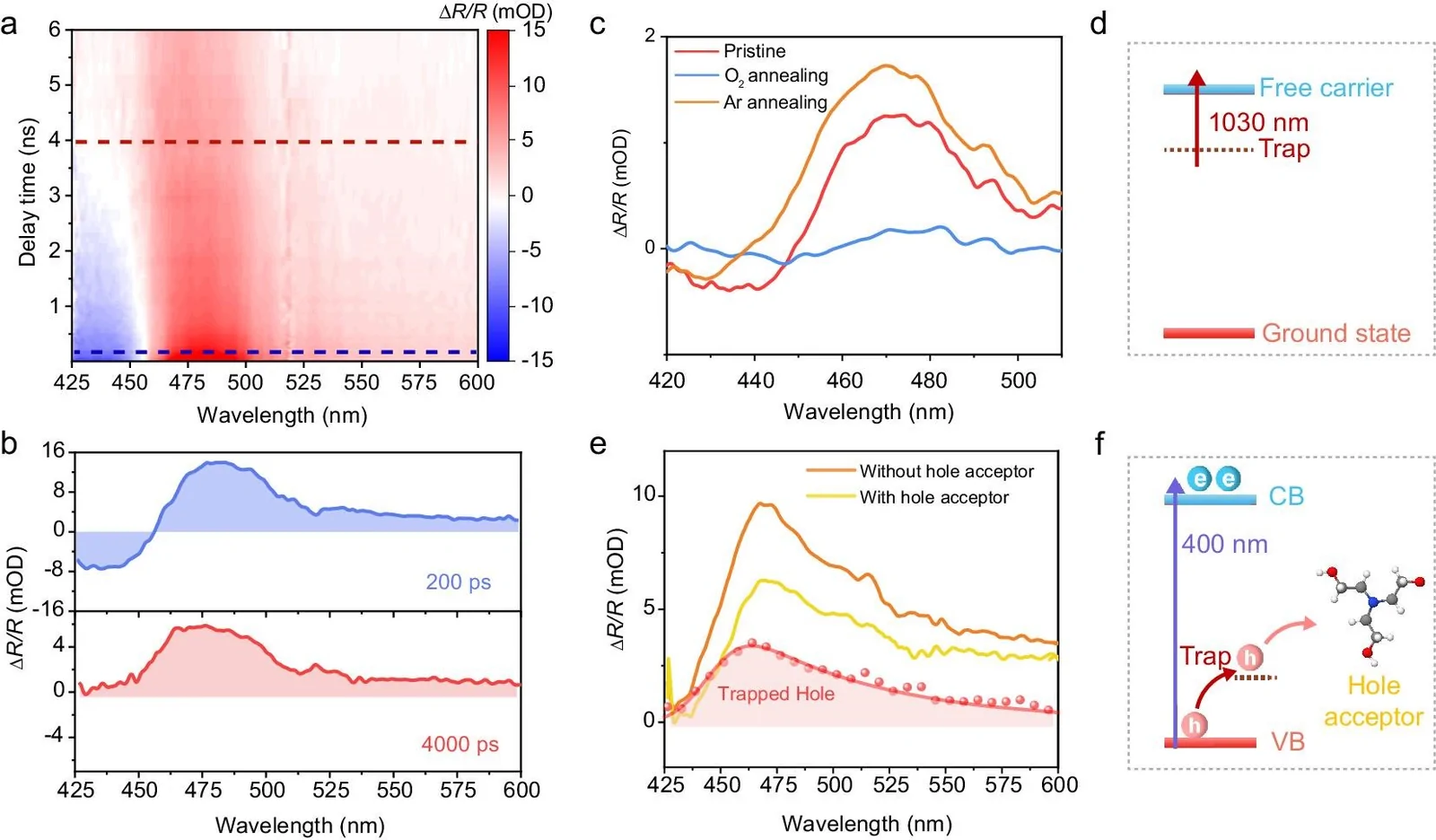
Transient reflection measurement of decahedral BiVO_4_ and the assignment of trapped hole signal. (a) 2D pseudo-color map of transient reflection spectra of decahedral BiVO_4_ measured on {110} facets. (b) Transient reflection spectra of decahedral BiVO_4_ measured on {110} facets at 200 and 4000 ps. (c) Transient reflection spectra of pristine, O_2_-annealed and Ar-annealed decahedral BiVO_4_ at 100 ps. (d) Schematic illustration of sub-bandgap excitation of trap state in decahedral BiVO_4_. (e) Transient reflection spectra of decahedral BiVO_4_ measured with and without TEOA as a hole acceptor at 3000 ps. The red line is the extracted trapped hole signal, exhibiting as a difference of the two spectra. (f) Schematic illustration of the hole extraction process in the presence of a hole acceptor for decahedral BiVO_4_.

In order to investigate the interfacial kinetics in the photocatalytic process, we employed an *in situ* TRM system to monitor the carrier dynamics on a single BiVO_4_ crystal from picoseconds to submilliseconds with ∼500 nm spatial resolution (Figs S3–S5). Decahedral BiVO_4_ crystals were hydrothermally synthesized onto a yttria-stabilized zirconia (YSZ) substrate, and then immersed in a thin layer of reaction solution (e.g. H_2_O or inert DMF (*N,N*-Dimethylformamide) solvent) for measurement. We first converted both the 400 nm femtosecond pump and the time‑delayed probe pulses to circular polarization to suppress the interference from optical anisotropy [28] (Fig. S6). The pump pulse then excited the crystal, and the probe pulse reflected from the sample–solution interface was collected by a fiber-optic spectrometer. Because YSZ and BiVO_4_ have a similar refractive index [29,30], reflections from the sample–substrate interface are suppressed, ensuring that the detected signal predominantly represents carrier dynamics at the sample–liquid interface where the photocatalytic reaction occurs [31]. Figure 2a and b show the transient reflection (TR) spectra recorded on the {110} facets in the inert solution. At early delay times, the TR spectra shape agrees well with our previous results on BiVO_4_ sample [9], which consists of two components: (i) a derivative-like signal near the bandgap region (∼2.7 eV) arising from transient bandgap shifts, and (ii) the valence-band hole signal described by the Drude model in the >500 nm region [32].

As the delay time increases, a new positive peak at 470 nm emerges and gradually dominates the spectra in the near bandgap region (Fig. 2b). To explore its origin, we carried out several control experiments, including sub-bandgap excitation and annealing treatment under different conditions. Sub-bandgap excitation at 1030 nm reproduced the characteristic peak at 470 nm (Fig. 2c), suggesting that it most likely arises from a trap state energetically near the conduction or valence band edges of BiVO_4_. Subsequently, BiVO_4_ crystals were annealed in O_2_ or Ar atmospheres, and TR spectra were compared (Fig. 2c). It is obviously observed that annealing in an O_2_ atmosphere significantly suppressed the characteristic peak at 470 nm, while slightly enhanced it in Ar environment, which implies that such trap states are closely related to the oxygen‑related species on the surface of BiVO_4_. X-ray photoelectron spectroscopy of the O 1s region (Fig. S7) reveals three components at 529.8, 530.6 and 531.6 eV corresponding to lattice oxygen, defective oxygen species and hydroxyl group, respectively [33,34]. It is noted that the intensity of defective oxygen species at 530.6 eV decreases after O_2_ annealing, while it remains unchanged after Ar annealing, further indicating that the trap state is contributed by the defective oxygen species intrinsically. Furthermore, density functional theory (DFT) results suggest that trap states could arise on the {110} surface with bridge-bonded oxygen vacancies (Bi–O_v_–Bi and Bi–O_v_–V), possibly corresponding to the oxygen-related trap state described above (Figs S8 and S9).

Furthermore, to determine whether the observed trap state captures photogenerated carriers, hole scavengers such as triethanolamine (TEOA) were introduced [35] and the TR spectra were compared (Fig. 2e). At a delay time of 3 ns, both the TR spectra collected with and without hole scavengers are dominated by the positive signal after excitation. The peak intensity at 470 nm is substantially quenched by adding hole scavengers, indicating an efficient hole extraction from the trap states, with kinetics occurring on a sub-nanosecond timescale (Fig. S10). Moreover, the possible contribution from electron signals to the trap signal was ruled out through introducing the electron scavenger (Fig. S11). By subtracting the characteristic signals with and without hole scavengers, we isolate the trap state contribution (red differential curve in Fig. 2e) and assign the feature at 470 nm to the photogenerated holes trapped at defective oxygen‑related sites. Based on the above results, the obtained TR spectra on the BiVO_4_ at the near bandgap region can be decomposed into two components: (i) a positive peak centered at 470 nm arising from trapped holes, and (ii) a derivative‑like feature reflecting transient refractive index changes at the band edge. Figure S12 shows the TR spectral decomposition of the BiVO_4_ crystal, with the derivative and trapped hole components plotted in blue and red, respectively. Besides, the robustness of TR spectral deconvolution was proved by the similarity between fitted trapped hole kinetics and kinetics extracted at the isosbestic point of the derivative signal as shown in Fig. S13.

**Figure 3.**
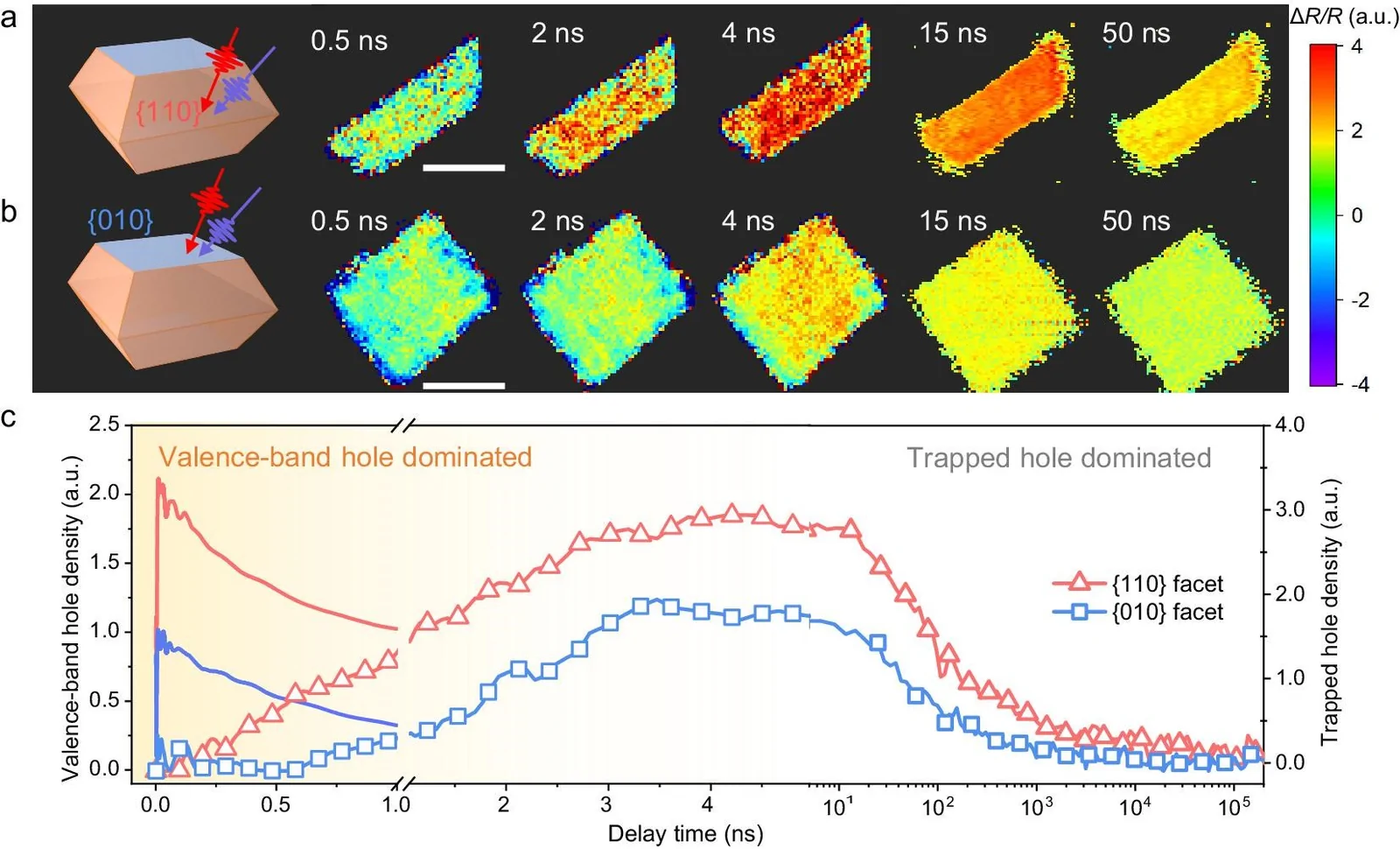
Spatial and temporal evolution of photogenerated holes of decahedral BiVO_4_ measured in inert solvent. Distribution of the trapped holes on the {110} (a) and {010} facets (b) of decahedral BiVO_4_ crystals at different delay times. (c) Temporal evolution of valence-band holes (solid lines) and trapped holes (dotted lines) on the {110} and {010} facets. Scale bars are 2 μm.

To visualize the spatiotemporal behavior of the trapped holes on the decahedral BiVO_4_ crystals, we performed pump-probe TR mapping from picoseconds to microseconds. The trapped-hole mapping was derived by subtracting a scaled image acquired at 430 nm (derivative-like signal dominated) from the image at 480 nm (trapped-hole dominated) (Figs S14 and S15). Figure 3a and b present the resulting maps on the {110} and {010} facets, respectively. In both cases, the signal of trapped holes gradually rises over 4 ns and then decays over the nanosecond to microsecond timescale. Notably, the trapped holes exhibited a pronounced spatial distribution, emerging at 500 ps and persisting throughout the entire time window. ​These signals preferentially accumulated on the {110} facets of BiVO_4_ crystals, consistent with the spatial charge-separation property supported by *in situ* photochemical deposition results in Fig. 1.

To quantify facet‑dependent charge carrier dynamics of BiVO_4_ crystals, we decomposed the TR spectra at each delay into valence-band hole and trapped hole contributions and extracted their kinetics on the {110} and {010} facets (Fig. 3c). The signal of valence-band holes appears as a broad negative signal from 550 to 800 nm (Fig. S16) [9,22], and its kinetics (solid lines) shows that more valence-band holes accumulate on the {110} facets, reaching their peak by ∼5 ps after photoexcitation. This facet-dependent charge-separation process aligns with the ultrafast, anisotropy‑driven charge separation reported in other semiconductor photocatalysts [8,9]. As the population of valence-band hole decays, the signal of trapped holes (dotted lines) concurrently rises with a single‑exponential growth time constant of ∼1.5 ns (Table S1), indicating first-order trapping kinetics. The subsequent power-law decay of trapped holes (Fig. S17) suggests a dispersive recombination characteristic of trapped carriers [36].

To confirm that this facet-dependent disparity in the trapped holes originates from early charge separation rather than from different defect densities, we performed sub-bandgap excitation at 1030 nm, which can probe the defect concentration directly. Statistical analysis of the resulting TR signal (Fig. S18) revealed a relatively higher defect concentration on the {010} than {110} facets. Furthermore, spatial mapping of the intraband photoluminescence (PL) emission from defect states demonstrates a significantly higher PL intensity on the {010} facets, providing further evidence for the facet-dependent defect distribution (Fig. S19). Overall, the defect distribution revealed by facet-dependent TR measurement and PL imaging shows a different trend from that of the trapped holes, ruling out the effect of defect heterogeneity.

### Trap-mediated interfacial hole transfer mechanism

**Figure 4.**
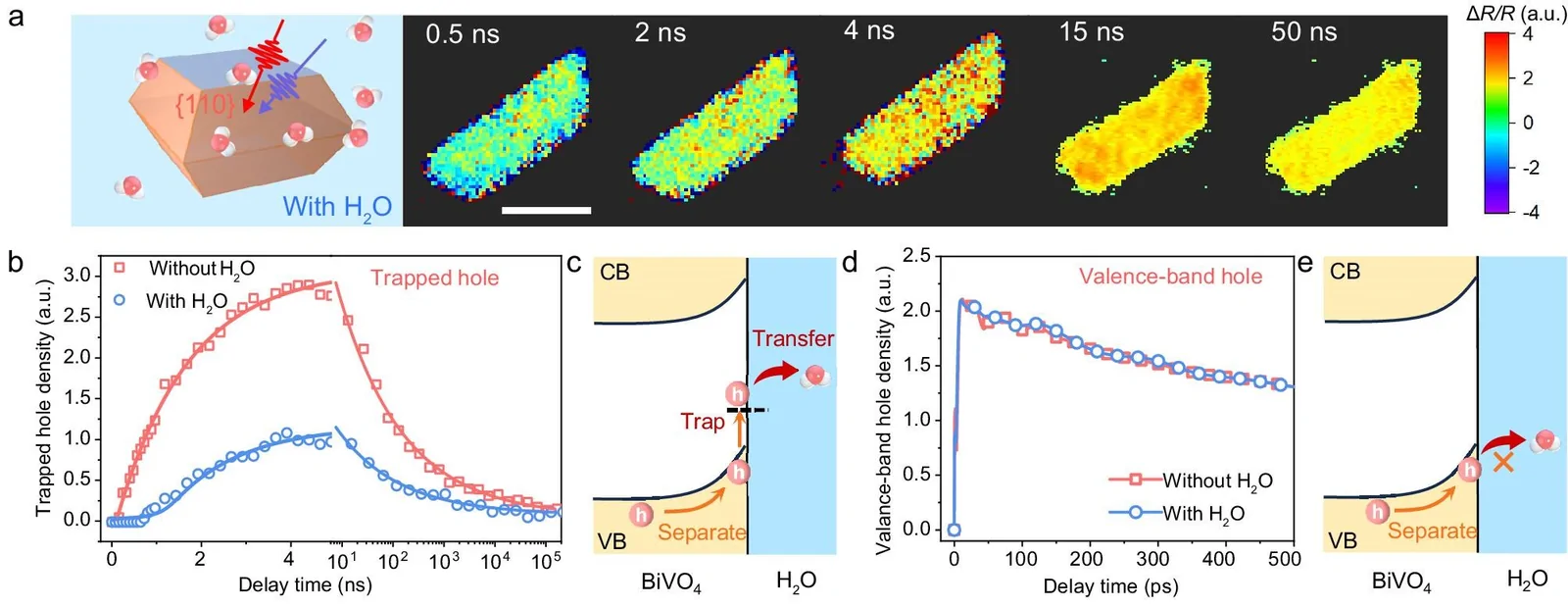
Spatial and temporal evolution of photogenerated holes of decahedral BiVO_4_ with and without introducing H_2_O molecules. (a) Distribution of trapped holes on the {110} facets measured in water at different delay times. (b and c) Dynamics of trapped holes on the {110} facets of BiVO_4_ measured with and without water, and schematic illustration of the proposed trapped hole transfer mechanism. The solid lines are the fitting results of the interfacial hole transfer model (details are in the Supplementary data) and power-law decay. (d and e) Dynamics of valence-band holes on the {110} facets of BiVO_4_ measured with and without water, and schematic illustration of the proposed valence-band hole transfer mechanism. Scale bar is 2 μm.

It is proposed that long-lived trapped holes are generally inert species incapable of driving subsequent photocatalytic processes [37–39]. However, charge carriers localized in shallow traps may still retain sufficient reactivity [40,41]. To assess the photocatalytic involvement of trapped holes, we conducted TR measurements in H_2_O solution instead of inert DMF to track their kinetics under realistic reaction conditions. As shown in Fig. 4a and Fig. S20, introduction of H_2_O causes a significant quenching of the trapped hole signal on both {110} and {010} facets, indicating rapid hole extraction by water. Because the refractive indeces of DMF (*n* = 1.43) [42] and H_2_O (*n* = 1.33) are close, this signal attenuation cannot be attributed to optical artifacts. The virtually unchanged early time TR spectra measured in water further validate this (Fig. S21).

Deconvolution of the TR spectra can yield the trapped hole kinetics in the presence of H_2_O. As shown in Fig. 4b, in contrast to the inert DMF solvent, introducing H_2_O molecules in the system facilitates rapid hole extraction with no detectable signal within the first nanosecond, suggesting that the ultrafast interfacial transfer process far exceeds the hole trapping timescale. Subsequently, the trapped hole population recovers slowly, ultimately reaching a lower concentration than in the inert solution. The unexpectedly high charge transfer rate suggests that the pre-adsorbed H_2_O molecules serve as the reacting species, as the mass-transfer rates would be significantly slower. Consequently, the subsequent increase in the trapped hole signal may originate from the depletion of these pre-adsorbed H_2_O molecules.

To verify this hypothesis, we varied the photocarrier density by changing the pump power and observed that at low power densities (∼10^19^ cm^−3^), trapped hole extraction is nearly complete, whereas higher densities leave an increasing fraction of holes unextracted, indicative of the limited concentration of pre-adsorbed H_2_O molecules (Figs S24 and S25). Additionally, reducing the water concentration progressively inhibits hole extraction (Fig. S26), further confirming that surface‑adsorbed H_2_O is the reactive species. Finally, to track the hole transfer kinetics under conditions more relevant to practical photocatalysis, we switched the pump source from a 400 nm femtosecond laser to a 450 nm continuous-wave laser (CW laser) at an intensity equivalent to ∼100 sun illumination. We achieved essentially complete hole extraction under this CW laser illumination (Fig. S27), demonstrating that under practical photocatalytic conditions where water coverage far exceeds photoinjected carrier density, the trapped holes can be efficiently and fully utilized (Fig. 4c). This efficient interfacial charge transfer may account for the prominent photocatalytic activity of the BiVO_4_ crystal.

To further elucidate the charge transfer mechanism, we investigated whether hole extraction proceeds directly from valence-band holes to H_2_O or occurs via intermediate trapping at surface states. To this end, we analyzed and compared the valence-band hole kinetics in inert and reactive solutions (Fig. 4d and Fig. S28). Their similarity excludes direct valence-band hole transfer to water (Fig. 4e), indicating that the oxygen-related trap states mediate the interfacial hole transfer. To probe the involvement of bond‑making or bond‑breaking steps, we further substituted H_2_O with isotopically labeled D_2_O and H_2_^18^O to assess the possible kinetic isotope effect [43]. No noticeable change was observed after isotope substitution (Fig. S29), suggesting that the ultrafast hole transfer process probed here does not involve the formation or cleavage of chemical bonds.

In order to quantify the hole transfer kinetics, we developed a simplified kinetic model (details are given in the Supplementary data), in which valence-band holes undergo first-order trapping to populate defect states, and trapped holes react with pre-adsorbed H_2_O molecules via a second-order process. The trapped hole kinetic traces were globally fitted using a least-square method, with the fitted results shown in Fig. 4b as solid lines. The extracted rate constant for hole transfer (*K_r_*) and the effective initial concentration of pre-adsorbed H_2_O concentration ([H_2_O_ads,0_]) were 13.1 × 10^−10^ cm^−3^ s^−1^ and 0.41 × 10^20^ cm^−3^, respectively. Under practical conditions, the trapped hole density is much lower than pre-adsorbed H_2_O concentration ([H_2_O_ads_]); therefore, the second-order reaction effectively follows pseudo-first-order kinetics, yielding an estimated hole transfer lifetime of ∼19 ps.

### Kinetic model for photogenerated hole evolution in photocatalysis

**Figure 5.**
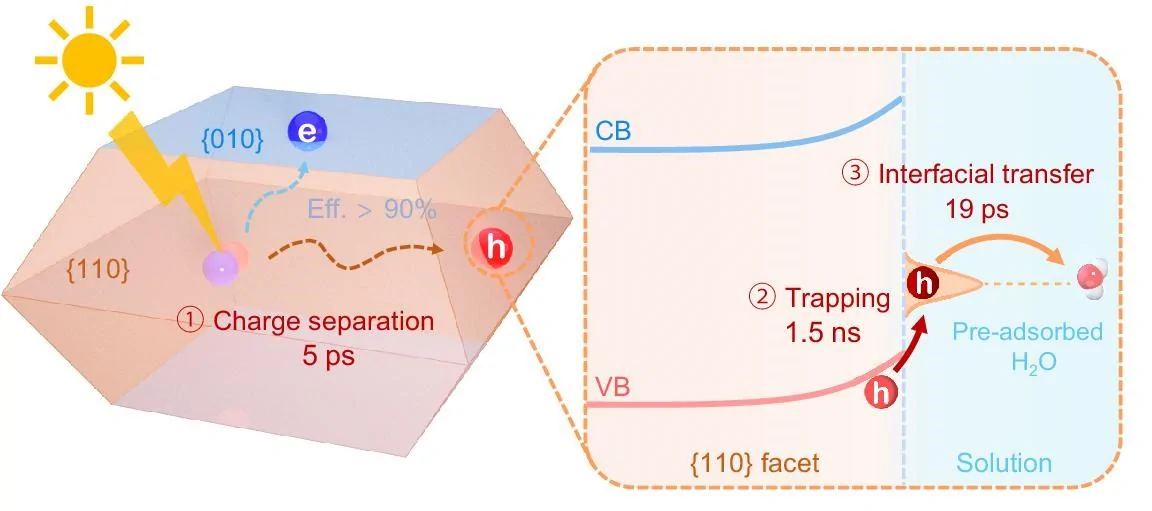
Schematic illustration of the proposed photogenerated hole evolution pathway involving three steps: ultrafast charge separation, hole trapping and rapid interfacial transfer.

Based on these findings, we propose a comprehensive model to describe the entire evolution of photogenerated holes in BiVO_4_ (Fig. 5), comprising three successive steps: (i) ultrafast photogenerated charge separation within 5 ps with holes accumulating on {110} facet; (ii) hole trapping in defect states induced by oxygen-related species at a time constant of ∼1.5 ns and (iii) rapid interfacial hole transfer mediated by oxygen-related defect at a time constant of ∼19 ps. Notably, the interfacial hole transfer rate observed here corresponds to a timescale that is significantly faster than the millisecond-to-second timescales reported previously [40,44,45]. Beyond intrinsic material differences, this discrepancy likely stems from two key factors. First, unlike common bulk-probing techniques (e.g. transient absorption and flash photolysis) [6,46], our surface-sensitive TRM (with a detection depth of tens of nanometers [9]) effectively decouples interfacial kinetics from slow bulk carrier transport, allowing direct tracking of the intrinsic charge transfer at the solid–liquid interface. Secondly, most investigations on interfacial transfer processes were performed by detecting intermediates in the photocatalysis cycle where new chemical bonds have already formed [47,48]. In contrast, our work only captures the initial transfer step at very early times, without involving the subsequent slow bond-breaking and bond-making processes. This likely explains the difference observed here.

Moreover, given defect engineering as a proven strategy to boost photocatalytic activity [49], the defect-mediated interfacial hole transfer likely plays a pivotal role in the prominent water-oxidation performance of BiVO_4_. On the one hand, the efficiency of photocatalysis is fundamentally limited by the rapid recombination of photogenerated electrons and holes. The rapid interfacial hole transfer observed here can effectively extract holes from the photocatalyst before recombination occurs, thereby significantly suppressing carrier recombination and minimizing associated energetic losses. On the other hand, the first hole transfer and the subsequent formation of the OH* intermediate are widely recognized as the rate-limiting steps in the sluggish oxygen evolution reaction (OER) [50]. The rapid transfer mechanism revealed in this work suggests that defect engineering may effectively lower the kinetic barrier for this critical initial step and facilitate sluggish elementary steps in OER, ultimately improving photocatalytic performance [51]. Based on the discussion above, this complete, real-time visualization of carrier evolution across the solid–liquid interface clarifies the functional role of trapped holes and highlights the essential contribution of surface defect states in enabling efficient photocatalytic oxidation reactions.

## CONCLUSION

In summary, we have established a complete, spatiotemporal map of photogenerated hole dynamics in decahedral BiVO_4_ crystals under realistic photocatalytic conditions. Shortly after photoexcitation, ultrafast charge separation quickly establishes a facet-dependent charge distribution within 5 ps, with holes located primarily on {110} facets. Subsequently, valence-band holes are captured by trap states with a characteristic trapping time of ∼1.5 ns. Crucially, these trapped holes act as nanoscale relay sites, enabling ultrafast interfacial transfer to pre-adsorbed H_2_O on the BiVO_4_ surface in ∼19 ps, whereas direct valence-band hole transfer without defect mediation is effectively negligible, which is the main reason for the high quantum efficiency of water oxidation on the BiVO_4_ photocatalyst. This nanoscale picture, linking facet geometry, atomic defect sites and interfacial chemistry, identifies trap engineering and facet control as practical nanoscale design rules for accelerating interfacial charge transfer and improving water-oxidation efficiency.

## Supplementary Material

nwag379_Supplemental_File
