# Supplementary material for: Unraveling the evolution of photogenerated holes from charge separation to interfacial transfer in photocatalysis
Source: Natl Sci Rev. 2026 Jun 18;13(17):nwag379. doi: 10.1093/nsr/nwag379 (PMC13563667; doi:10.1093/nsr/nwag379)
Supplement: Supplementary file 1 [file nwag379_supplemental_file.pdf]

Supplementary Data For:

Unraveling the evolution of photogenerated holes from charge separation to interfacial transfer in photocatalysis

Fengke Sun[1,3,†], Yue Zhao[2,†], Xianchang Yan[4], Panwang Zhou[2], Can Li[2], Rengui Li[2*], Shengye Jin[1] and Wenming Tian[1*]

[1]State Key Laboratory of Chemical Reaction Dynamics, Dalian Institute of Chemical Physics, Chinese Academy of Sciences, Dalian 116023, China;

[2]State Key Laboratory of Catalysis, Dalian National Laboratory for Clean Energy, iChEM (Collaborative Innovation Center of Chemistry for Energy Materials), Dalian Institute of Chemical Physics, Chinese Academy of Sciences, Dalian 116023, China

[3]University of Chinese Academy of Sciences, Beijing 100049, China and [4]ZJU-Hangzhou Global Scientific and Technological Innovation Center, Zhejiang University, Hangzhou 311200, China

***Corresponding authors.** E-mails: rgli@dicp.ac.cn; tianwm@dicp.ac.cn

[†]Equally contributed to this work.

Preparation of $BiVO_4$ Crystal Seed Layer on YSZ substrate

1 mmol of $Bi(NO_3)_3 \cdot 5H_2O$ and 1 mmol of NH_3VO_3 were dissolved together in 60 mL of 2 M nitric acid solution under 60 °C. Subsequently, 2 g of polyvinyl alcohol was introduced into mixture and stirred until fully dissolved. The resulting solution was then drop-cast (25 μL per 1.0 × 2.0 cm^2 substrate) onto pre-cleaned YSZ substrate. The seed layer was fabricated by spin-coating at 1000 rpm for 60 s, followed by calcination in air at 450 °C for 2 h.

Synthesis of Decahedral $BiVO_4$ Crystals

0.1 mmol of $Bi(NO_3)_3 \cdot 5H_2O$ and 0.1 mmol of NH_3VO_3 were dissolved in 60 mL of 2 M HNO_3 and stirred at 40°C until fully dissolved. The pH of the solution was then adjusted to 1.10 using ammonia solution (25–28 wt.%) under vigorous stirring, and the mixture was stirred for an additional 30 min. The solution was transferred to a 100 mL Teflon-lined stainless-steel autoclave containing a piece of $BiVO_4$-seeded YSZ substrate at the bottom. Hydrothermal growth was carried out at 200°C for 12 h. After naturally cooling to room temperature, the substrates were rinsed with ultrapure water and dried under an air stream.

Synthesis of $BiVO_4$ nanoparticles

$BiVO_4$ nanoparticles were prepared by two different methods:

hydrothermal synthesis (denoted as nanoparticle-HT) and solid-state-reaction synthesis (denoted as nanoparticle-SSR) for comparison.

Hydrothermal synthesis: 10 mmol of $Bi(NO_3)_3 \cdot 5H_2O$ and 10 mmol of NH_3VO_3 were dissolved in 60 mL of 2 M nitric acid solution under continuous stirring until a clear solution was obtained. The pH of the solution was then carefully adjusted to 4.0 using 25-28 wt.% aqueous ammonia under vigorous stirring, followed by continued stirring for an additional 2 hours. The resulting solution was transferred into a 100 mL Teflon-lined stainless-steel autoclave and subjected to hydrothermal treatment at 200°C for 24 hours in an electric oven. After naturally cooling to room temperature, a yellow precipitate was collected by centrifugation, thoroughly washed with ultrapure water, and dried at 80 °C overnight.

Solid-state synthesis: In a typical synthesis procedure, 2.0 mmol of $Bi(NO_3)_3 \cdot 5H_2O$ and 1.0 mmol of V_2O_5 were mixed by grinding in an agate mortar until a homogeneous mixture was obtained. The resulting powder was then calcined in a muffle furnace at 823 K for 10 h. After cooling to ambient temperature, the product was collected for characterizations.

Photo-reduction deposition of noble metals

100 mg of $BiVO_4$ powder and 0.5 wt% $HAuCl_4$ metal precursor was dispersed in 100 mL of deionized water. The suspension was irradiated with a 300 W xenon lamp under continuous stirring. After 30 minutes of

photo-deposition, the suspension was filtered, washed thoroughly with deionized water for three times, and dried at 60°C overnight.

Photo-oxidation deposition of MnO_x

100 mg of $BiVO_4$ powder and 1 wt% $MnSO_4$ precursor was dispersed in 100 mL of 5 mM $NaIO_3$ solution. The suspension was irradiated with a 300 W xenon lamp under continuous stirring for 3 hours. The resulting suspension was then filtered, washed thoroughly with deionized water for three times, and dried at 60°C overnight.

Femtosecond-Resolved Transient Reflection Microscopy (fs-TRM)

Transient reflection spectra and imaging on the picosecond to nanosecond timescale of $BiVO_4$ crystal were conducted on our home-built femtosecond-resolved transient reflection microscope (fs-TRM) system, as shown in Figure S3. The system was based on a TA100 transient absorption system from Dalian Time-Tech Spectra Co, Ltd, and some modifications were made to meet our requirements. To be specific, a Yb:KGW laser (1030 nm, 100 kHz repetition rate, 100 μJ per pulse, 200 fs) from Hangzhou Yacto Technology Co., Ltd was selected as the light source. 90% of the light pulse was used to pump our home-built nonlinear optical parametric amplifier (NOPA) to generate a pump pulse. In this experiment, 400 nm was selected as the pump wavelength, and the energy density to excite the sample was set to $6.6*10^{19}$ cm^{-3} unless otherwise stated. The rest

10% light pulse was used to generate supercontinuous white light as the probe pulse. For the short wavelength white light, the 1030 nm pulse first passed a β-BBO crystal (2 mm) to generate a second harmonic 515 nm pulse, then the 515 nm pulse was focused on a sapphire crystal (3 mm) to generate a white light ranging from 420 nm to 500 nm. For the long wavelength white light, the 1030 nm pulse was directly focused on a YAG crystal (13 mm) to generate white light ranging from 520 nm to 950 nm. The pump pulse was modulated by a chopper (MC2000B-EC, Thorlabs, Inc) at a repetition rate of 1kHz. Then, the pump and probe pulses were combined by a longpass dichroic mirror and sent into microscope system (IX73, Olympus Co. Ltd). For the TR spectra measurement, the collinear pump and probe pulses were focused onto the $BiVO_4$ crystal surface immersed in DMF/H_2O as inert/reactive medium by an oil-immersion 100 ×objective (NA=1.45, UPLXAPO100XO, Olympus). The reflected probe pulse was collected back through the same objective and finally brought to focus. The probe pulse was then collected by an optical fiber (diameter = 100 μm, NA = 0.22) and dispersed by a monochromator equipped with 150 grooves/mm grating. A CMOS linear image sensor was used to collect the reflected probe pulse. For TR imaging measurement, both the pump and probe pulses were defocused before oil-immersion 100 × objective to achieve wide-field illumination with a spot size of ~15 μm. The reflected probe pulse was also collected by the objective and recorded by an area

scan CMOS sensor (acA720-520um, Basler).

Nanosecond-Resolved Transient Reflection Microscopy (ns-TRM)

Transient reflection spectra and imaging on nanosecond to sub-millisecond timescale $BiVO_4$ crystal were conducted on a home-built nanosecond-resolved transient reflection microscope (ns-TRM) system (Figure S4). A Ti-Sapphire Laser (800 nm, 1 kHz, 6 mJ, 35 fs) from Coherent Co. Ltd. was selected as light source. The 800 nm laser passed a BBO crystal to generate a second harmonic 400 nm pulse and was selected as the pump pulse. A white light pulse was generated by a synchronized supercontinuum white laser (LEUKOS DISCO-2, 2 kHz). 90% of the white light pulse was used as probe pulse while the rest 10% light pulse was sent into reference detector to calibrate the laser intensity fluctuation. The delay between pump and probe pulse was controlled by a time delay generator (PCIe-6612, National Instruments). To correct for timing jitter, the time delay was recalculated by a Pendulum CNT-91 frequency analyzer. This approach ensures that the temporal resolution is maintained at approximately 1 ns[1]. The pump and probe pulses were combined by a longpass dichroic mirror and sent into microscope system (IX73, Olympus Co. Ltd). For TR spectra and imaging measurement, the colinear pump and probe pulses were defocused before oil-immersion 100 × objective to achieve wide-field illumination with a spot size of ~15 μm and avoid laser-

induced damage to the sample. The TR spectra and mapping were collected by the same optical fiber spectrometer and area-scan CMOS sensor used in fs-TRM.

Continuous-Wave Laser Excited Transient Reflection Microscopy

To track hole dynamics under practical photocatalytic reaction conditions, a 450 nm continuous-wave laser was selected as the pump pulse. The experimental configuration followed the previously described ns-TRM setup, except for a change in the pump pulse (Figure S5). Voltage pulses (frequency = 1 kHz, rising/falling edges <4 ns) were generated by an arbitrary wave function generator (Tektronix AFG31002) and used to trigger both the pump and probe pulse. The time delay between the two pulses was controlled by a time-delay generator, as mentioned in ns-TRM. Moreover, the 450 nm pump pulse was operated with 200 μs ON-time within each 1 ms cycle (20% duty cycle). The excitation power density was set to about 100 sun to guarantee a sufficient signal-to-noise ratio.

Characterization of Photocatalysts

The morphologies and particle sizes were examined by scanning electron microscopy (SEM, Quanta 200 FEG, FEI). The crystal structure of the $BiVO_4$ samples was characterized by X-ray powder diffraction (XRD) using a Rigaku D/Max-2500/PC powder diffractometer. Powder

samples were scanned with Cu-Kα radiation at 40 kV and 200 mA. XRD patterns were recorded over the 2θ range of 8-60° at a scan rate of 5°/min and a step size of 0.02°. Raman spectra were acquired using a Renishaw Raman spectrometer with a spectral resolution of 2 cm^{-1}. A 532 nm laser line from a solid-state laser was employed as the excitation source. The laser power at the sample was approximately 0.3 mW. X-ray photoelectron spectroscopy (XPS) was conducted X-ray photoelectron spectrometer (Thermofisher Escalab 250 Xi+, Thermofisher).

Evaluation of Photocatalytic Performance.

The photocatalytic O_2 evolution reactions were carried out in a closed gas circulation and evacuation system using a 300-W Xe lamp (Ushio-CERMAX LX300) and optical cutoff filter (kenko, L42; $\lambda \geq 420$ nm). 100 mg $BiVO_4$ powders was dispersed in 100 mL $Fe(NO_3)_3$ solution (10 mM) in a Pyrex reaction cell and thoroughly degassed by evacuation in order to drive off the air inside. The amount of evolved O_2 was determined by an on-line gas chromatograph (Shimadzu GC-8A, TCD, Ar carrier).

Measurement of Apparent Quantum Efficiency (AQE)

The AQEs were measured using a closed gas circulation and evacuation system and a 300-W Xe lamp (Ushio-CERMAX LX300) with a 365 nm, 420 nm, 460 nm, 480 nm, 500 nm band-pass filter (ZBPA 365, 420, 460,

480, 500, Asahi Spectra Co., FWHM: 10 nm). For each measurement, 200 mg of photocatalyst was dispersed in 150 mL of 4.0 mM Fe^{3+} aqueous solution in a Pyrex reaction cell (charge separation efficiency was assessed by adding 20% v/v methanol to the solution.). The number of incident photons reaching the solution was measured using a calibrated Si photodiode (LS-100, EKO Instruments Co., LTD.). The AQE (ϕ) was calculated using the following equation:

$$\phi(\%) = (A \cdot r \cdot N_A / I) \times 100$$

where A represents a coefficient (1 for Fe^{2+} evolution; 4 for O_2 evolution), r represents the evolution rate of Fe^{2+} or O_2, N_A represents Avogadro constant (6.02×10^{23} mol^{-1}), I represents the number of photons absorbed by the reaction solution.

Post-annealing of $BiVO_4$ samples

Post-annealing treatment was conducted in vacuum tube furnace (Yi Si Technology Co. Ltd.). Prior to heating, the tube furnace was purged by pure gas (O_2 or Ar) three times to ensure complete air removal. A continuous flow of the selected gas was then maintained throughout the experiment. The furnace heated to 500°C with a ramping rate of 20°C/min and then hold for 2 hours. Finally, the furnace cooled down naturally.

Computational Details.

All density functional theory (DFT) calculations were performed within the projector-augmented wave (PAW) framework[2] using the Vienna Ab initio Simulation Package (VASP)[3]. The Perdew–Burke–Ernzerhof (PBE)[4] exchange–correlation functional within the generalized gradient approximation (GGA) was adopted. A kinetic-energy cutoff of 520 eV was used for the plane-wave basis set. The atomic positions were optimized until the residual forces on all atoms were smaller than 0.02 eV^{-1}.

The Brillouin-zone integrations were carried out using Monkhorst–Pack k-point meshes of 6 × 6 × 3 for geometry optimization and 8 × 8 × 4 for the density-of-states (DOS) calculations of bulk $BiVO_4$. To explore surface electronic structures, a $BiVO_4$ (110) slab containing twelve $BiVO_4$ units was constructed, separated by a vacuum region of 20 Å to eliminate spurious interactions between periodic images. Three representative oxygen-vacancy configurations were examined: (i) an oxygen atom coordinated to two Bi atoms, (ii) one bridging Bi and V, and (iii) one bonded to two V atoms. For each defective surface, both structural optimization and DOS calculations were conducted using k-point meshes of 3 × 4 × 1 and 5 × 8 × 1, respectively. Dispersion interactions were included via the DFT-D3 scheme with zero damping[5].

Spatial-Resolved Photoluminescence (PL) Measurement

We used a home-built photoluminescence scanned imaging microscopy

coupled with a spectrometer and a time-correlated single photon counting (TCSPC) module to collect the PL spectum and intensity imaging of octahedral $BiVO_4$ sample. Excitation of the sample was achieved by a 405 nm pulsed laser (PIXEA-405, Aurea Technology, France). The laser was sent to 2D galvo mirror scan system to achieve x-y plane scanning and brought to focus by a 100 x oil-immersion objective (NA = 1.45, UPLXAPO100XO, Olympus Co.Ltd) onto the $BiVO_4$ crystal surface. The PL emission was then collected by the same objective. For spectra measurement, the PL emission was sent to the spectrometer with a 450 nm longpass filter to remove the laser. For imaging measurement, the PL emission was sent to TCSPC module and a 610-680 nm bandpass filter was used to selectively collect intraband PL emission from defect states of $BiVO_4$.

Calculation of Trapped Hole Mapping

For fs-TRM measurement (Figure S7), the 480 nm kinetics reflect both trapped holes and derivative signals, whereas 430 nm kinetics arise solely from derivatives. Therefore, trapped hole mapping can be obtained by removing derivative signal contribution in 480 nm kinetics based on the derivative component spectra and mapping at 430 nm.

In ns-TRM measurements, the trapped hole signal dominates the spectrum at long delay times, with only a minor contribution from the derivative component. Therefore, we select 470 nm as the probe

wavelength for trapped hole mapping.

Optical Anisotropy and the Removal of Electron Signal in TR Spectra

Based on our previous work, the transient reflectance (TR) spectra of $BiVO_4$ in the near-bandgap region primarily contain two components[6]: (1) a first-derivative feature arising from band edge shift[7], and (2) an electron polaron signal appearing as a positive peak centered at 455 nm. In this study, we identify an additional trapped hole signal that manifests as a similar positive peak at 470 nm, significantly complicating spectral interpretation.

Polarization-dependent TR measurements provide an effective approach to distinguish these overlapping signals. As shown in Figure S5a-b, the probe polarization dramatically alters the TR spectra, while varying the pump polarization shows negligible effects. This optical anisotropy aligns with steady-state measurements[8] and DFT calculations[9-10] for monoclinic $BiVO_4$, originating from its low-symmetry crystal structure and anisotropic electronic transitions.

At early delay times (e.g., 10 ps in Figure S5), the spectral features are dominated by the derivative component and electron polaron signal, with minimal contribution from the nanosecond-scale hole trapping process. The polarization difference spectrum (Figure S5c, green curve) reveals a peak at 455 nm, indicating that the polarization dependent TR

spectra may stem from the polarization response of electron signal. To further confirm the origin of optical anisotropy in TR spectra, Au was photodeposited onto decahedral $BiVO_4$ to extract photogenerated electrons. As shown in Figure S5c, removing the electron signal from the TR spectra via Au loading eliminated observable optical anisotropy in the Au/$BiVO_4$ sample, confirming that the anisotropy primarily arises from polarization-sensitive electrons near the bandgap region. A detailed investigation of electron polaron signal (Figure S5d-S5e) suggests that the electron polaron signal could be either positive or negative peak depending on the probe polarization, whereas a circular polarized probe could hardly detect the electron polaron. This polarization dependence may originate from the polaronic nature[11] this electron signal. Moreover, a gradual increase in kinetics corresponding to hole trapping process could be observed at nanosecond timescale, indicating that trapped hole signal could still be detected by a circular probe pulse.

Therefore, we use circular polarized pump and probe pulse to eliminate the electron polaron contribution, ensuring that the TR spectra in the near bandgap region simply consists of derivative component and trapped hole signal.

The Trap Mediated Interfacial Hole Transfer Model

The evolution of photogenerated holes consists of steps including

charge separation, trapping and interfacial transfer. Given that initial charge separation proceeds within 5 ps, it's rational to neglect this separation process, which proceeds much faster than subsequent trapping and interfacial transfer. Moreover, it's hypothesized that the mass transfer of pre-adsorbed water molecule can be omitted within the nanosecond timescale. Therefore, a simple kinetic model was proposed to estimate the interfacial hole transfer rate:

$$\frac{d[h^+]}{dt} = -k_{tr} * [h^+] \tag{1}$$

$$\frac{d\left[h^+_{trap}\right]}{dt} = k_{tr} * [h^+] - k_r * [H_2O_{ads}] * \left[h^+_{trap}\right] \tag{2}$$

$$\frac{d[H_2O_{ads}]}{dt} = -k_r * [H_2O_{ads}] * \left[h^+_{trap}\right] \tag{3}$$

where $[h^+]$ is the valence-band hole concentration; $\left[h^+_{trap}\right]$ is the concentration of trapped holes; $[H_2O_{ads}]$ is the effective concentration of pre-adsorbed H_2O molecules on the $BiVO_4$ surface. k_{tr} and k_r represent the first-order hole trapping rate constant and second-order interfacial hole transfer rate constant, respectively. A home-made program based on least square method was performed to fit the hole transfer rate k_r and concentration of effective pre-adsorbed H_2O molecules $[H_2O_{ads}]$. The initial concentration of valence-band hole $[h^+]_0$ was simply set as total photoinjected carrier density and the hole trapping rate constant k_{tr} was determined via fitting the kinetics measured in inert solution. Furthermore, the hole transfer rate k_r and effective initial concentration of pre-

adsorbed H_2O molecules $[H_2O_{ads,0}]$ were extracted thorough fitting the kinetics measured in water. Under realistic conditions (carrier density ~ 10^{15} cm^{-3}), the trapped hole density would be negligible compared to $[H_2O_{ads}]$, effectively reducing the second-order hole transfer reaction to pseudo-first-order kinetics. The apparent rate constant can be derived as the product of $[H_2O_{ads,0}]$ and k_r.

As a simplified model, it provides a straightforward method to evaluate the timescale of the interfacial hole transfer, and the acquired 19 ps time constant underscores the significant role of trap states in mediating efficient hole transfer.

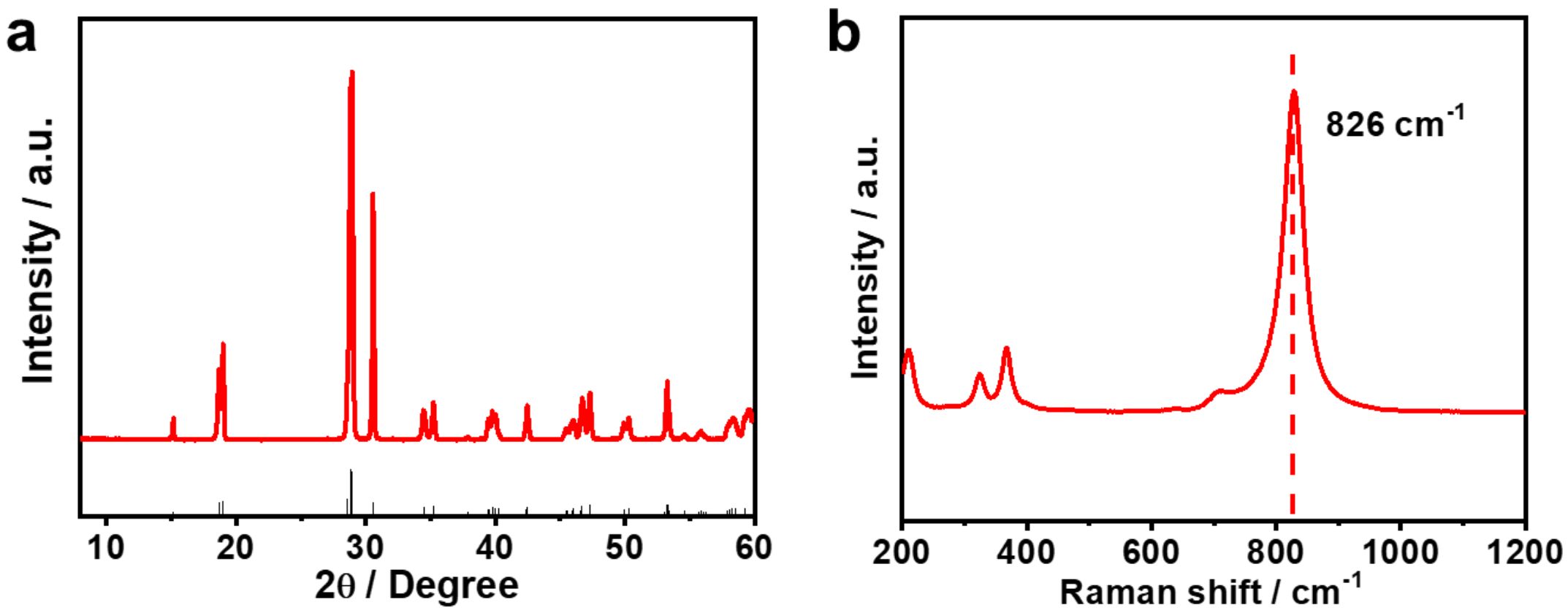

Figure S1. **a,** XRD pattern of decahedral $BiVO_4$ sample. **b**, Raman spectra of decahedral $BiVO_4$ sample. Raman peak at 826 cm^{-1} is assigned to symmetric V-O stretching mode in $BiVO_4$.

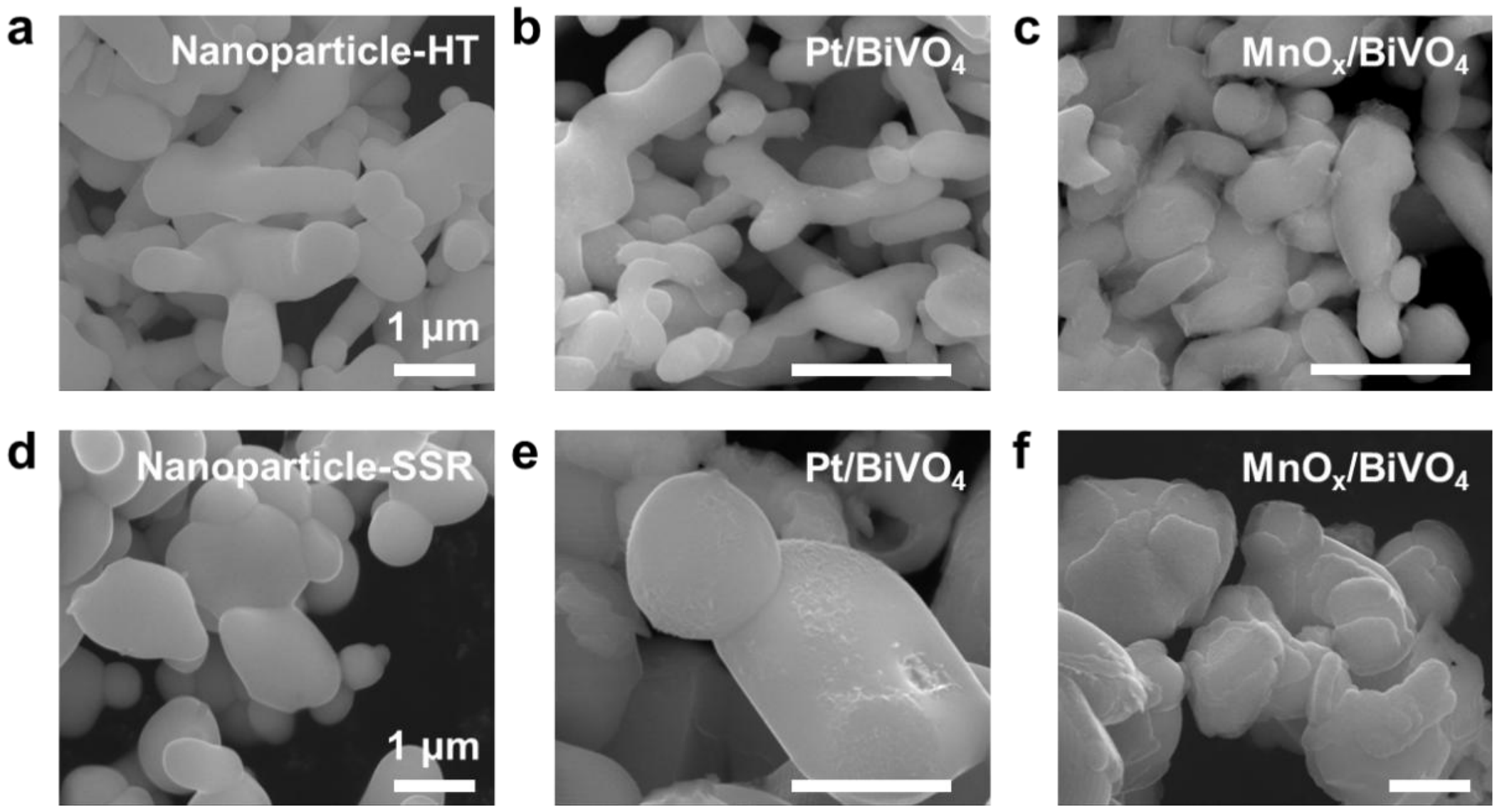

Figure S2. Morphology and photodeposition results of $BiVO_4$ nanoparticles. **a-c**, SEM images of nanoparticle-HT: **a**, pristine sample, **b**, after photoreduction deposition of Pt, and **c,** after photooxidation deposition of MnO_x. **d-f**, SEM images of nanoparticle-SSR: **d**, pristine sample, **e,** after photoreduction deposition of Pt, and **f,** after photooxidation

deposition of MnO_x. Both Pt and MnO_x are randomly distributed on the surface, suggesting the random distribution of photogenerated electrons and holes.

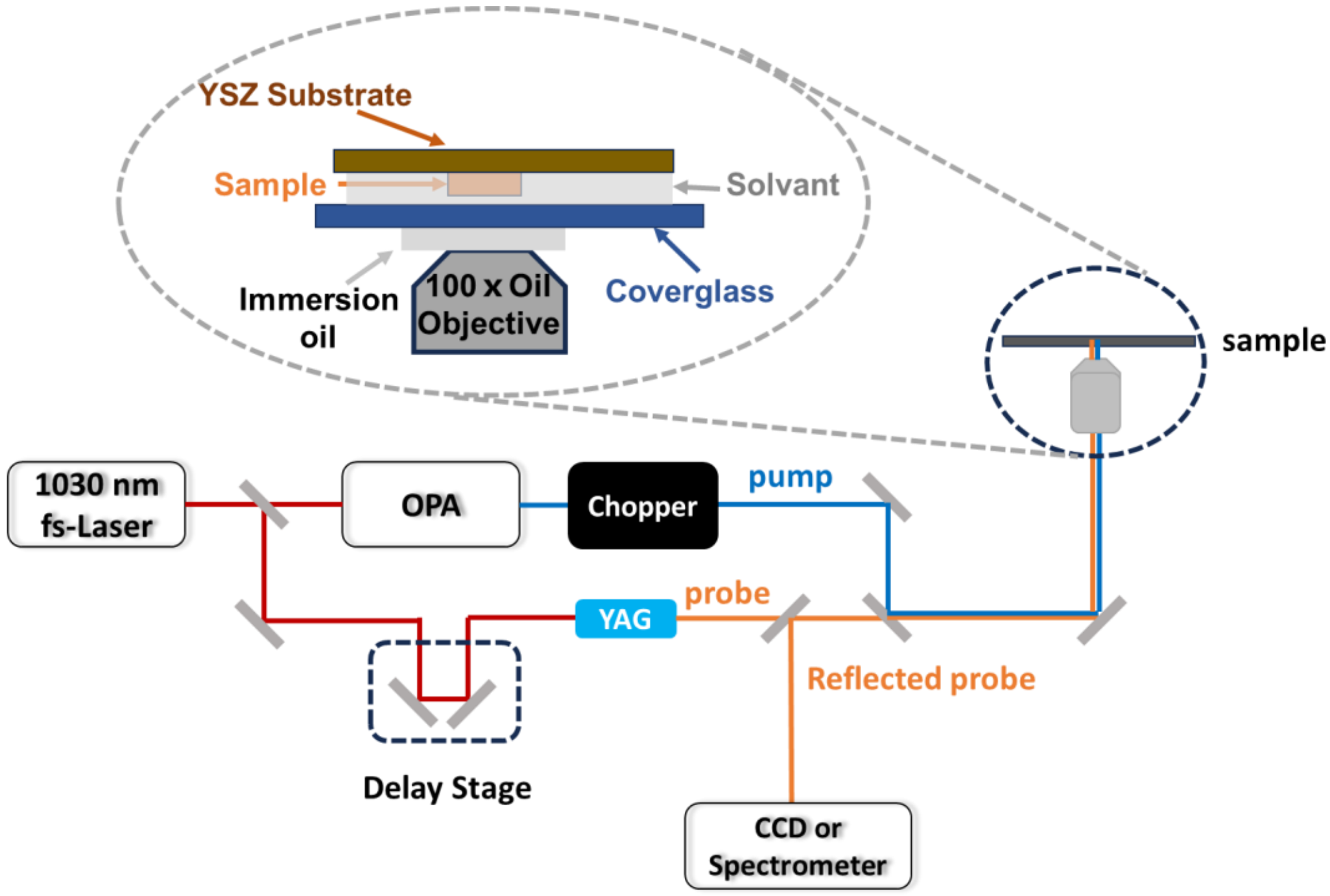

Figure S3. Schematic illustration of femtosecond-resolved transient reflection microscopy, OPA-optical parametric amplifier.

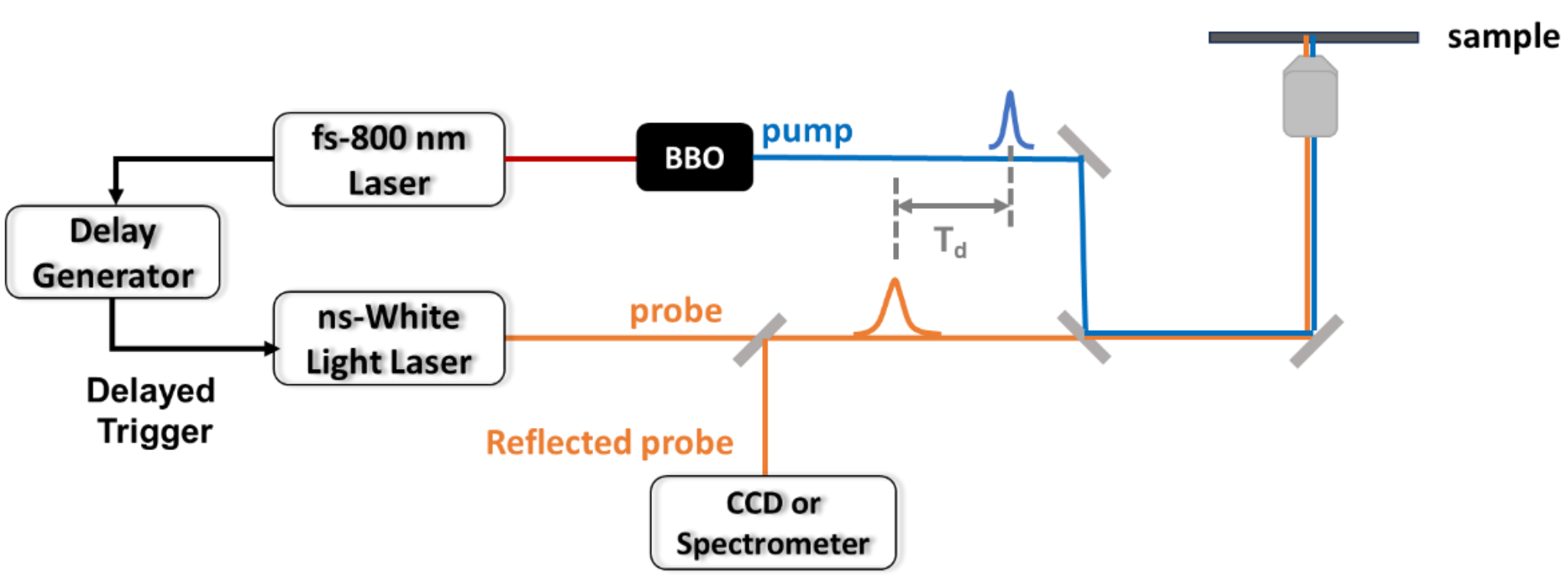

Figure S4. Schematic illustration of nanosecond-resolved transient reflection microscopy.

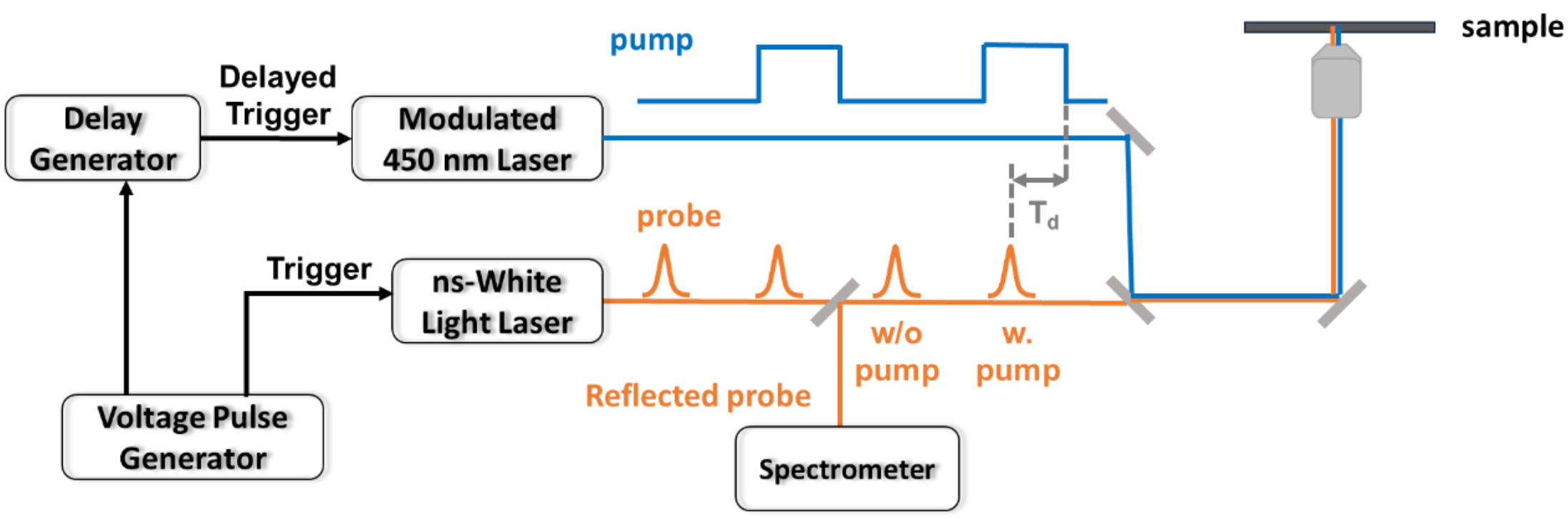

Figure S5. Schematic illustration of continuous-wave laser excited transient reflection microscopy.

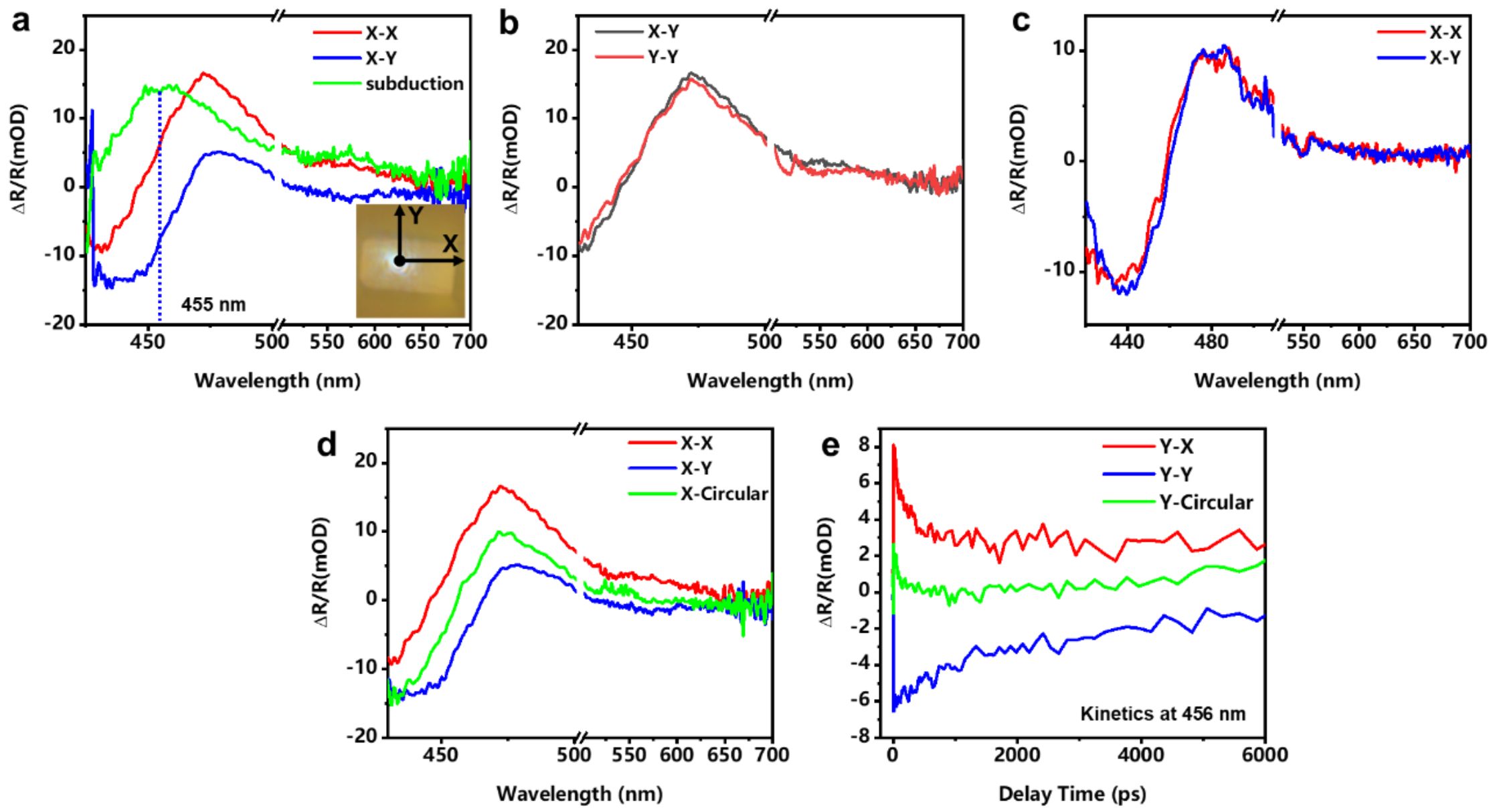

Figure S6. Polarization-dependent TR spectra of decahedral $BiVO_4$ on {010} facets. **a,** TR spectra at 10 ps measured under different probe polarizations. The inset illustrates the experimental coordinate system defining the polarization direction. The curve labels in figure represent the pump-probe polarization used in the experiment. For example, X-X denotes that both pump and probe light are linearly polarized along X-axis. **b,** TR spectra at 10 ps measured under different pump polarizations. **c,** TR

spectra at 1000 ps of Au/$BiVO_4$ sample measured under different probe polarizations. **d,** TR spectra at 10 ps measured under three different probe polarizations: X-polarized, Y-polarized, and circular polarized. **e,** TR kinetics at the isobestic point (456 nm, 2.7 eV) measured under three different probe polarizations.

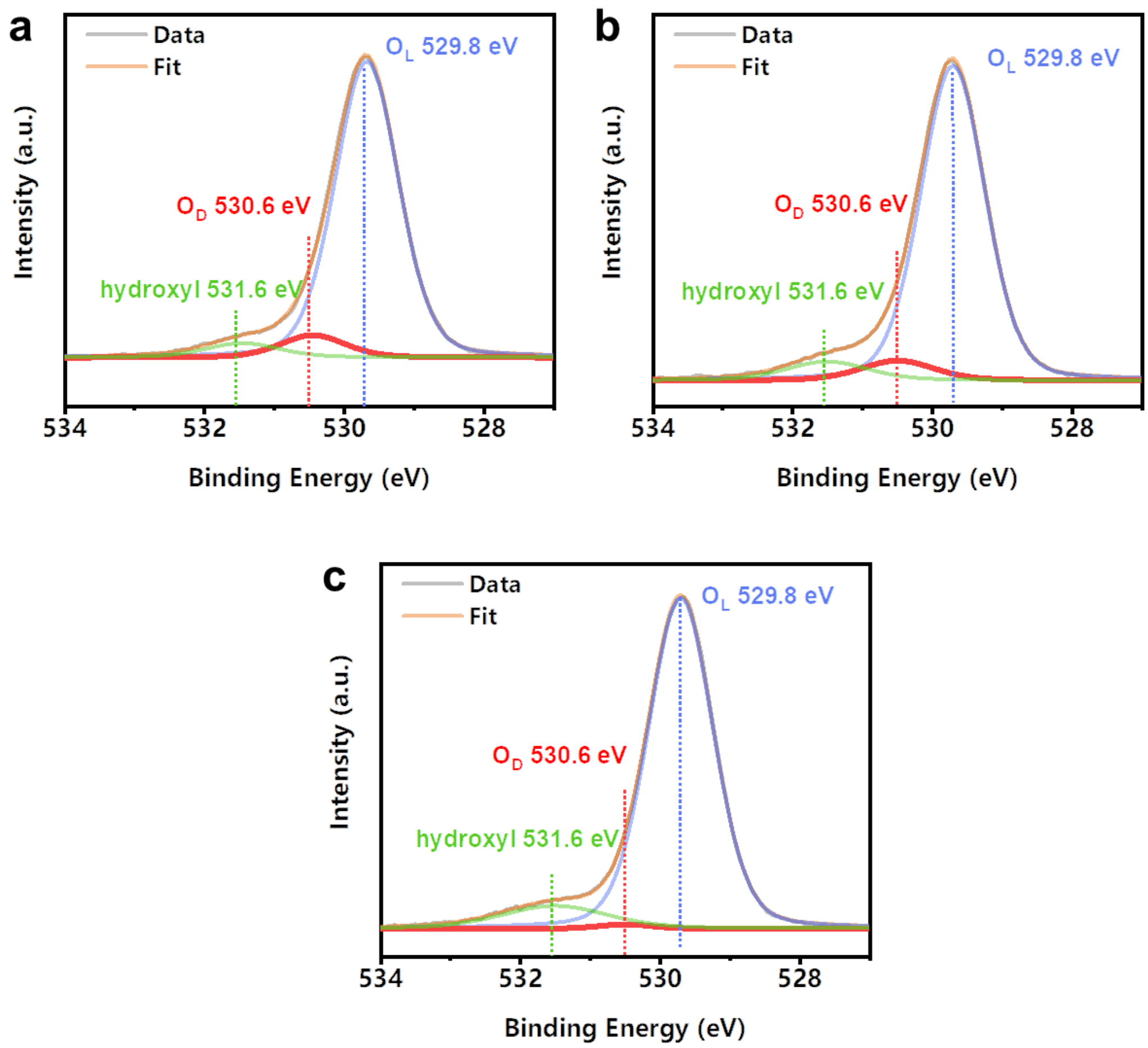

Figure S7. The O1s XPS spectra of $BiVO_4$ under 500°C Ar annealing (**a**), pristine $BiVO_4$ (**b**) and $BiVO_4$ under 500°C O_2 annealing samples (**c**). O_L, O_D and hydroxyl denote lattice oxygen, defective oxygen and surface absorbed hydroxyl groups.

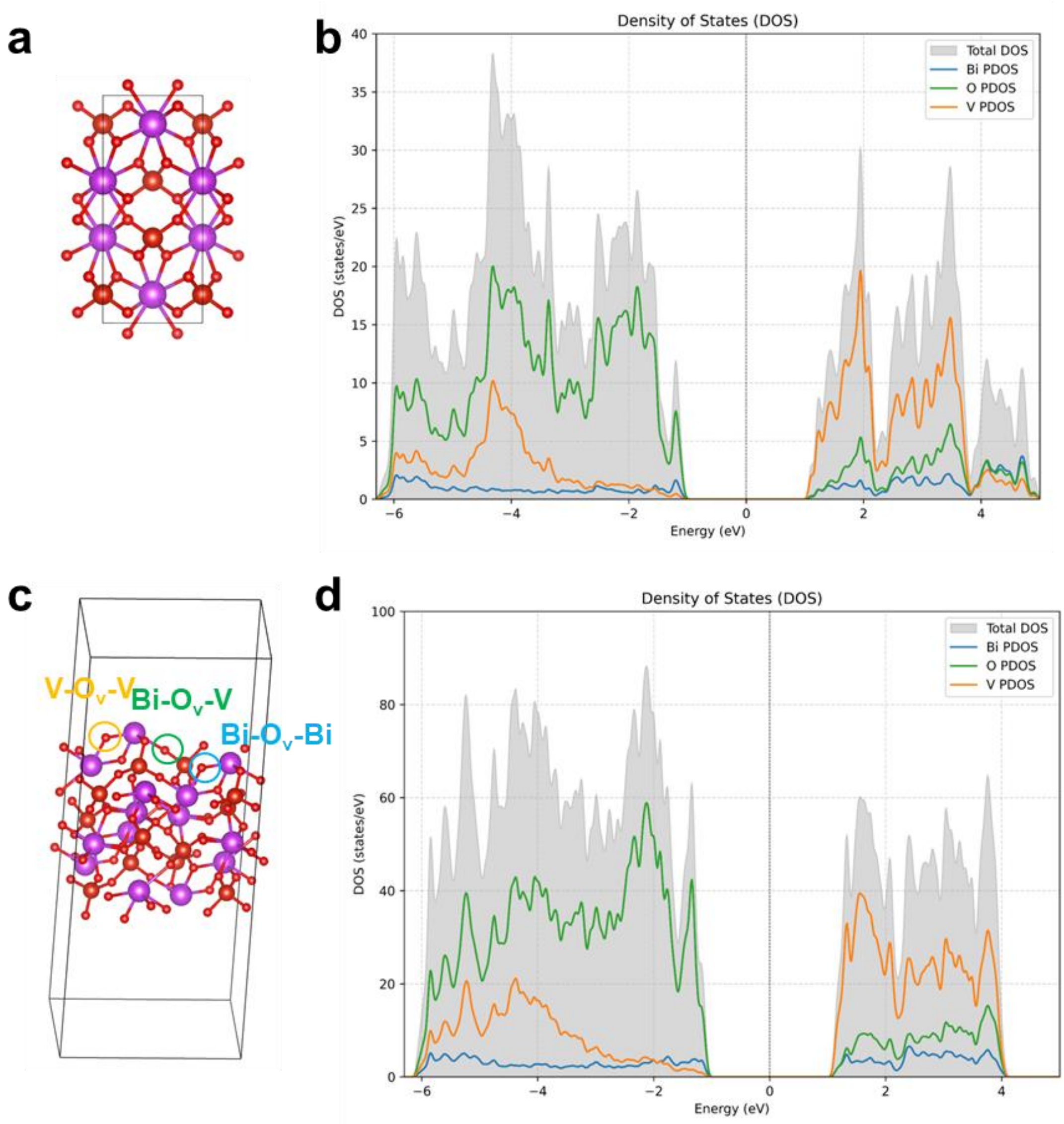

Figure S8. DFT calculation results of pristine $BiVO_4$. Crystal structure (**a**) and corresponding total (gray) and projected (colored) (density of states) DOSs (**b**) for bulk $BiVO_4$. Crystal structure (**c**) and corresponding total (gray) and projected (colored) DOSs (**d**) for $BiVO_4$ {110} surface. Purple spheres are Bi atoms, larger red spheres are V atoms, and small red spheres are O atoms. Three possible surface oxygen vacancies are also shown.

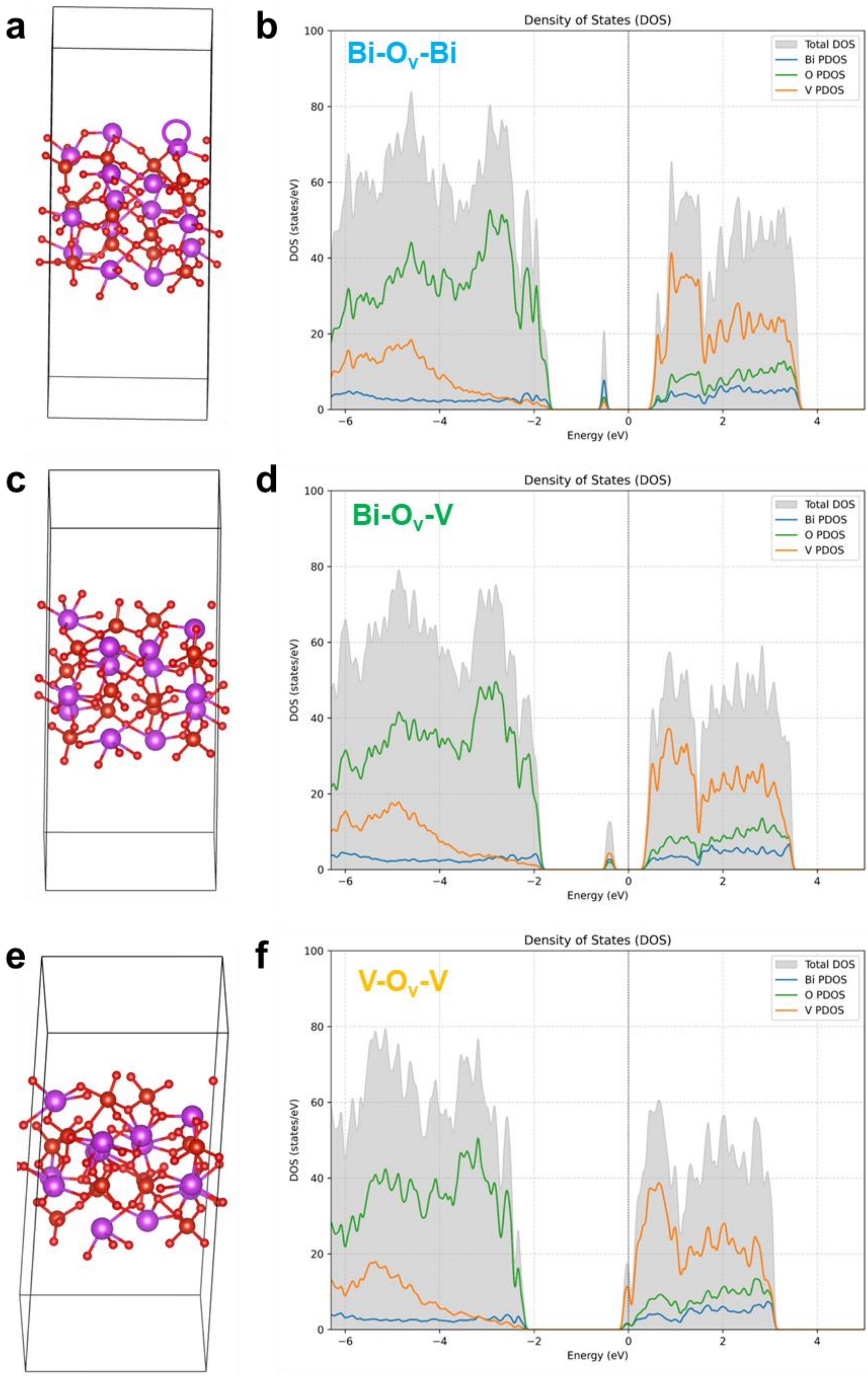
a
b
Density of States (DOS)
Bi-Ov-Bi
Total DOS
Bi PDOS
O PDOS
V PDOS
DOS (states/eV)
Energy (eV)
c
d
Density of States (DOS)
Bi-Ov-V
Total DOS
Bi PDOS
O PDOS
V PDOS
DOS (states/eV)
Energy (eV)
e
f
Density of States (DOS)
V-Ov-V
Total DOS
Bi PDOS
O PDOS
V PDOS
DOS (states/eV)
Energy (eV)

Figure S9. DFT calculation results for defective $BiVO_4$ with three different surface oxygen vacancy configurations. Crystal structure (**a**) and corresponding total (gray) and projected (colored) DOSs (**b**) for $BiVO_4$ with Bi-O_v-Bi surface oxygen vacancies. Crystal structure (**c**) and corresponding total (gray) and projected (colored) DOSs (**d**) for $BiVO_4$ with Bi-O_v-V surface oxygen vacancies. Crystal structure (**e**) and corresponding total (gray) and projected (colored) DOSs (**f**) for $BiVO_4$ with V-O_v-V surface oxygen vacancies. Only Bi–O_v–Bi and Bi–O_v–V configurations contribute to trap states within bandgap.

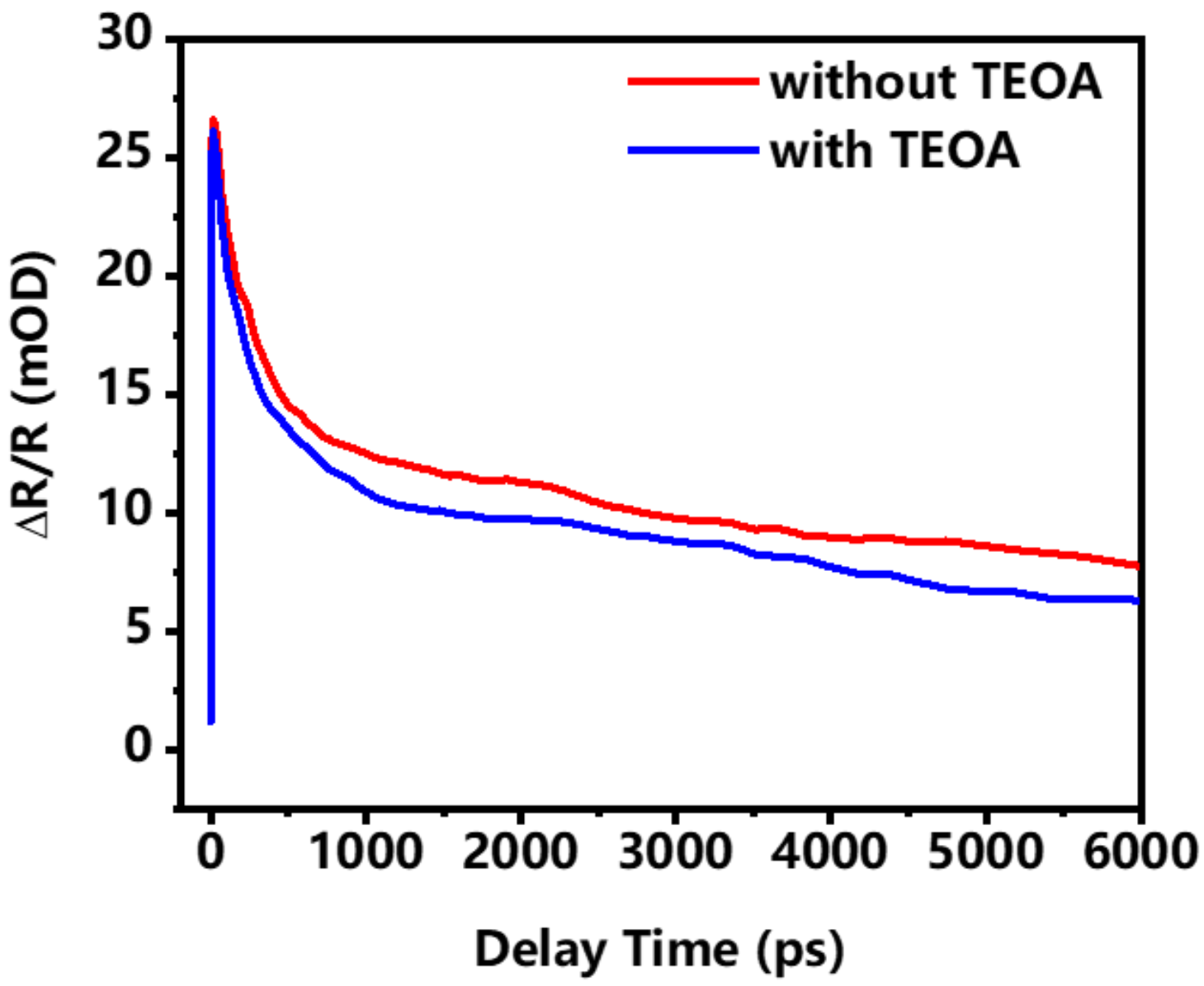

Figure S10. Comparison of TR dynamics at 470 nm of decahedral $BiVO_4$ measured with/without adding TEOA as hole acceptors.

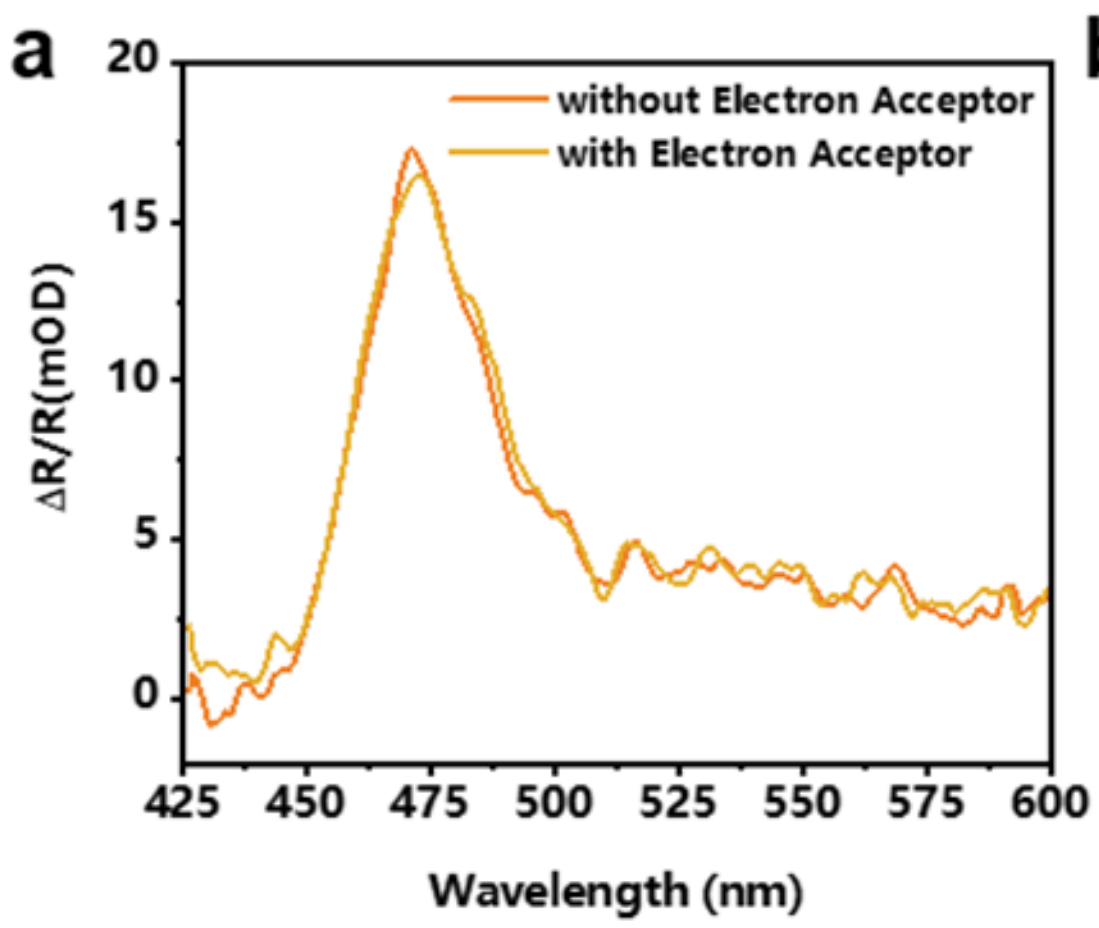

Figure S11. (a) TR spectra of decahedral $BiVO_4$ measured with and without 1,4-BQ as electron acceptor at 6 ns. TR kinetics at 470 nm measured with and without 1,4-BQ as electron acceptor.

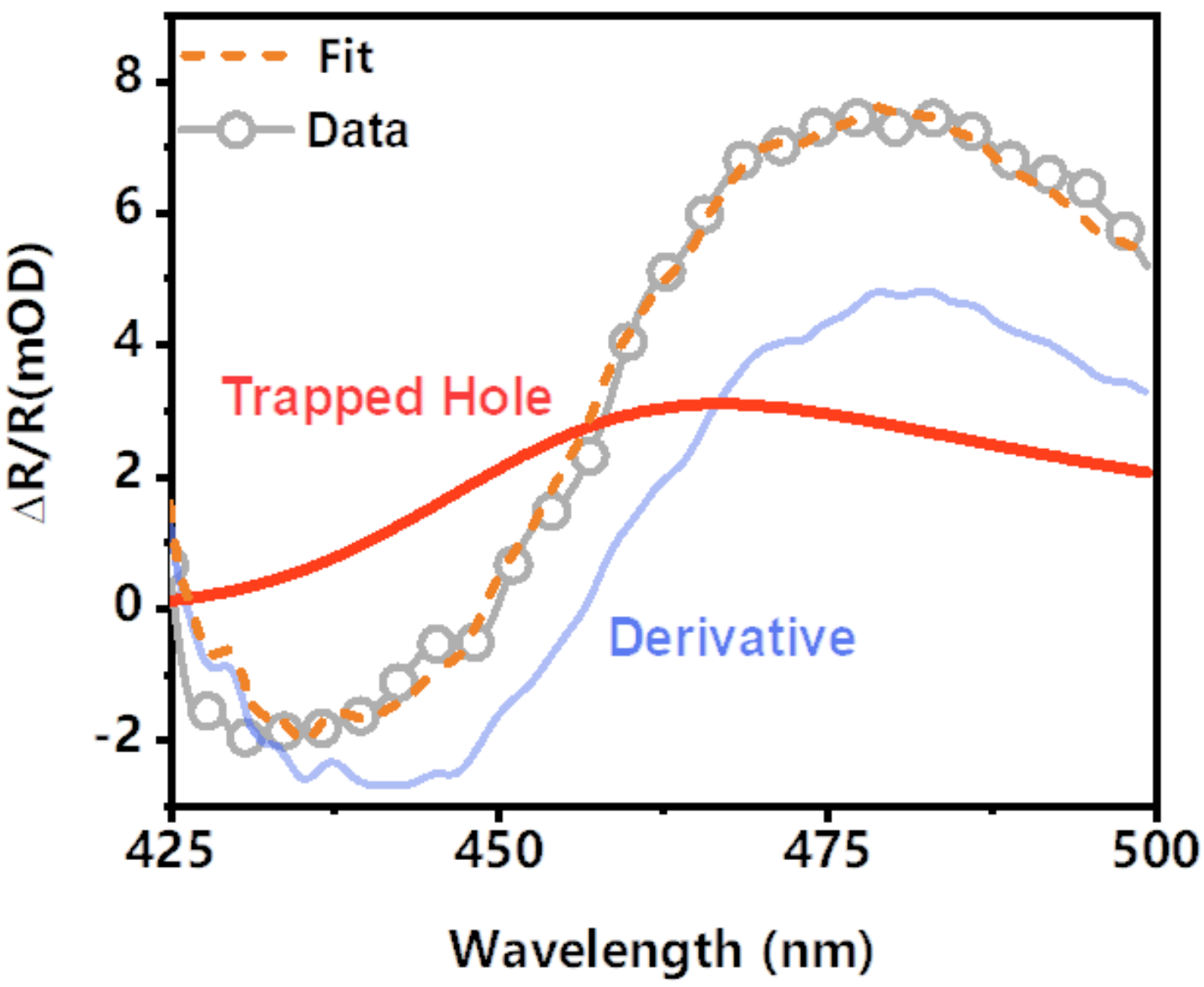

Figure S12. Transient reflection spectra in the near bandgap region and the decomposition result at 2000 ps. Blue and red lines represent the first derivative and trapped hole contribution, respectively. The gray circle is the original data, and the orange line is the fit of the transient spectra by these two components.

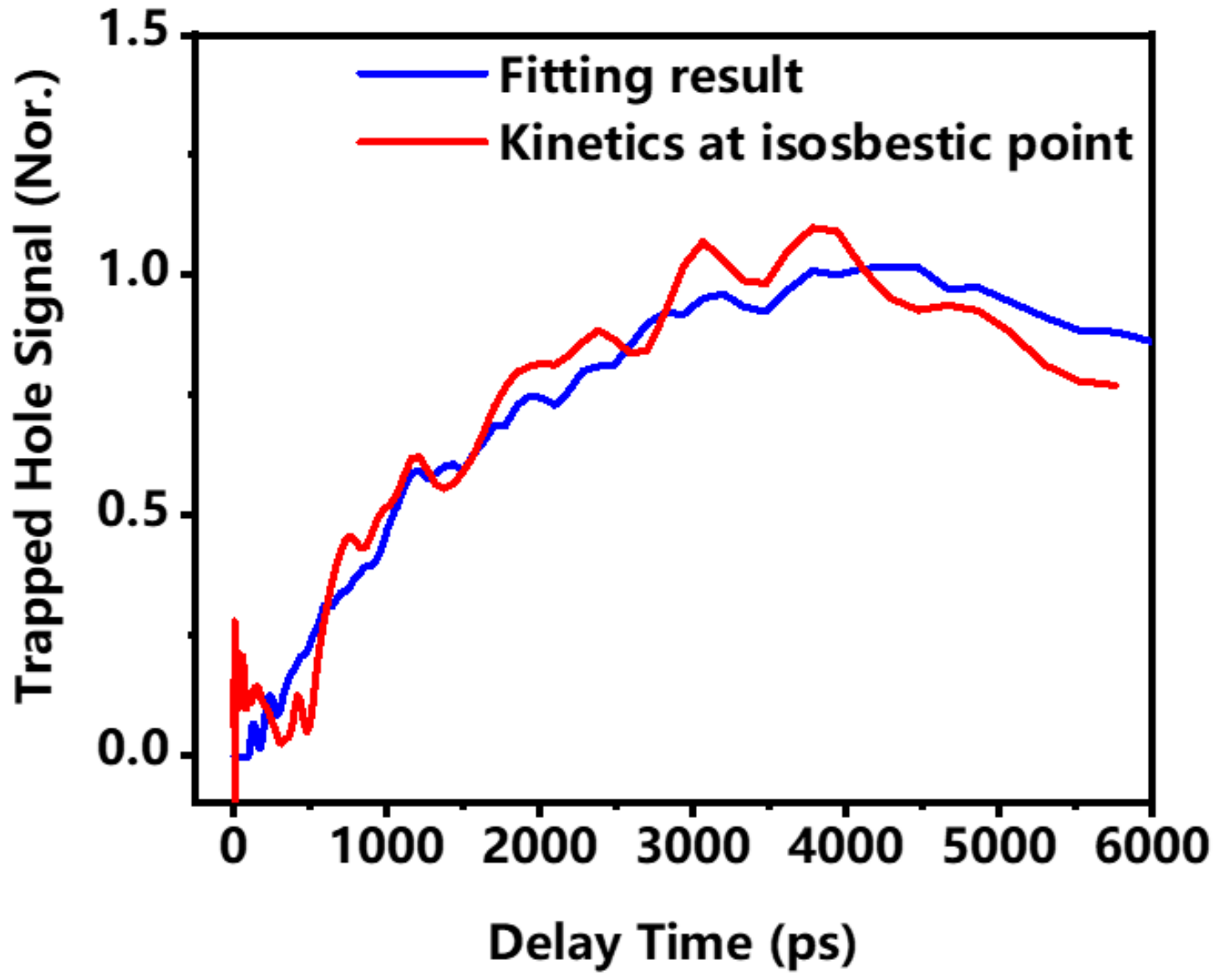

Figure S13. Comparison of trapped hole kinetics from fitting and extracting at isosbestic point.

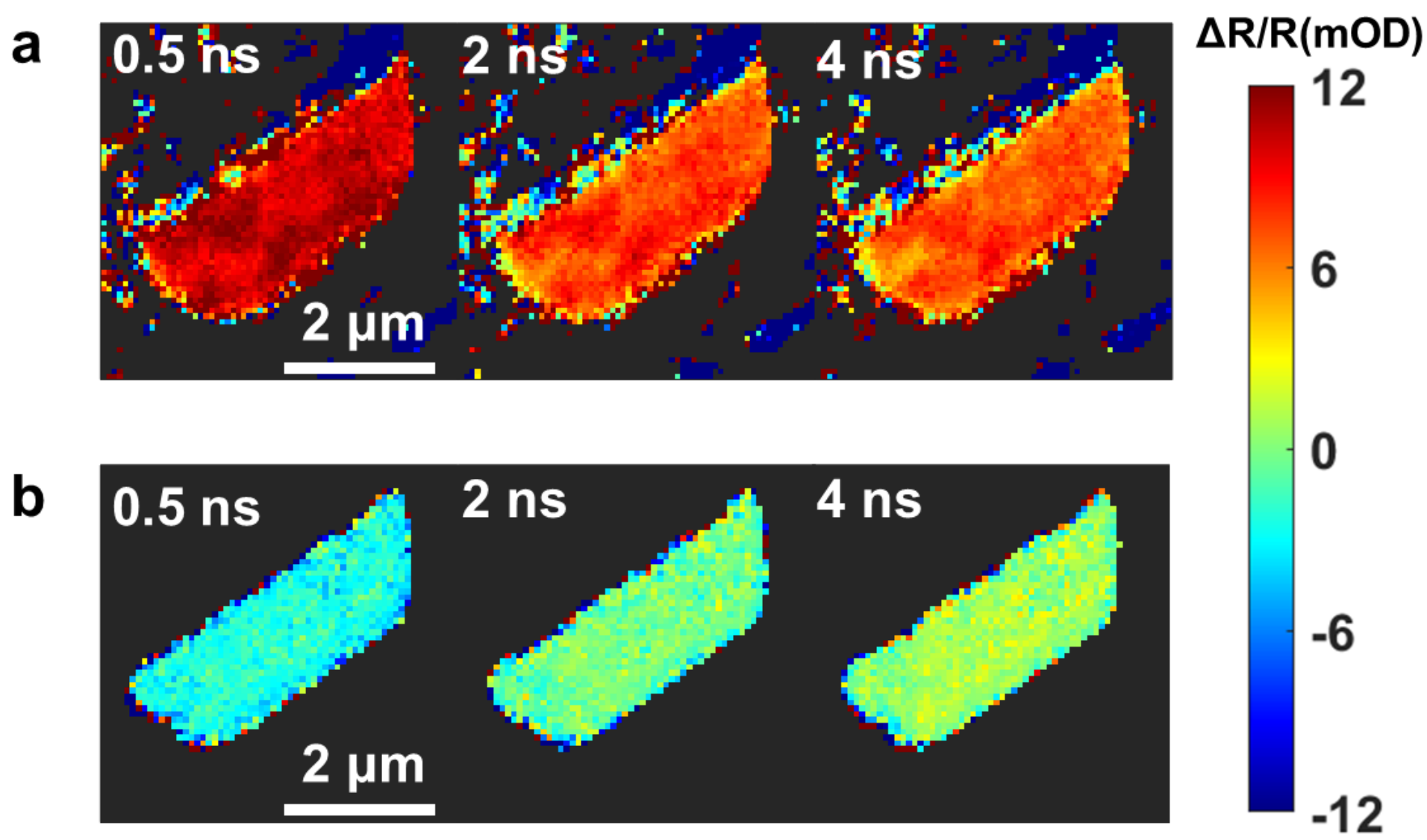

Figure S14. fs-TRM mapping on {110} facets of decahedral $BiVO_4$ in DMF at 480 nm (**a**) and 430 nm (**b**) at different delay times.

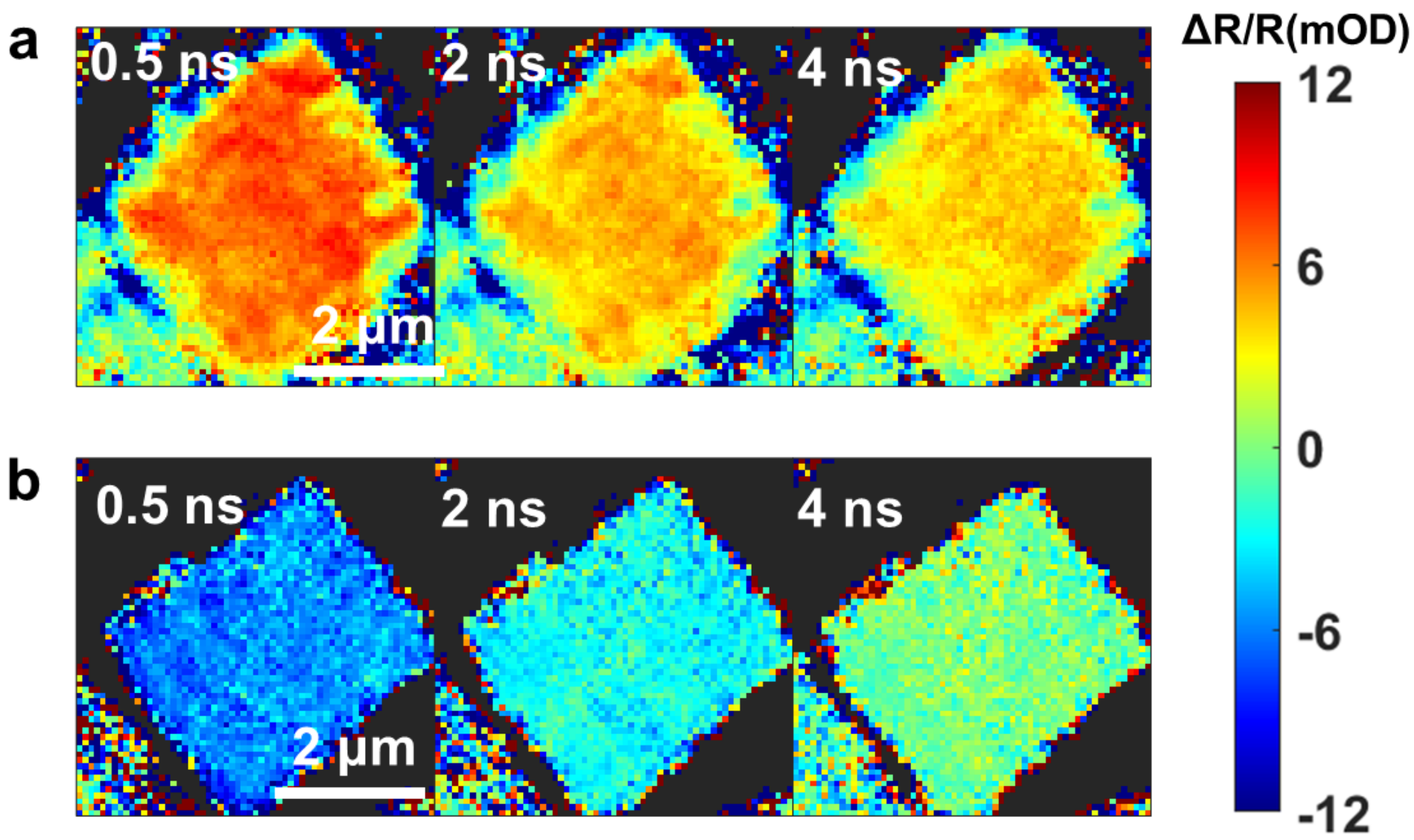

Figure S15. fs-TRM mapping on {010} facets of decahedral $BiVO_4$ in DMF at 480 nm (**a**) and 430 nm (**b**) at different delay times.

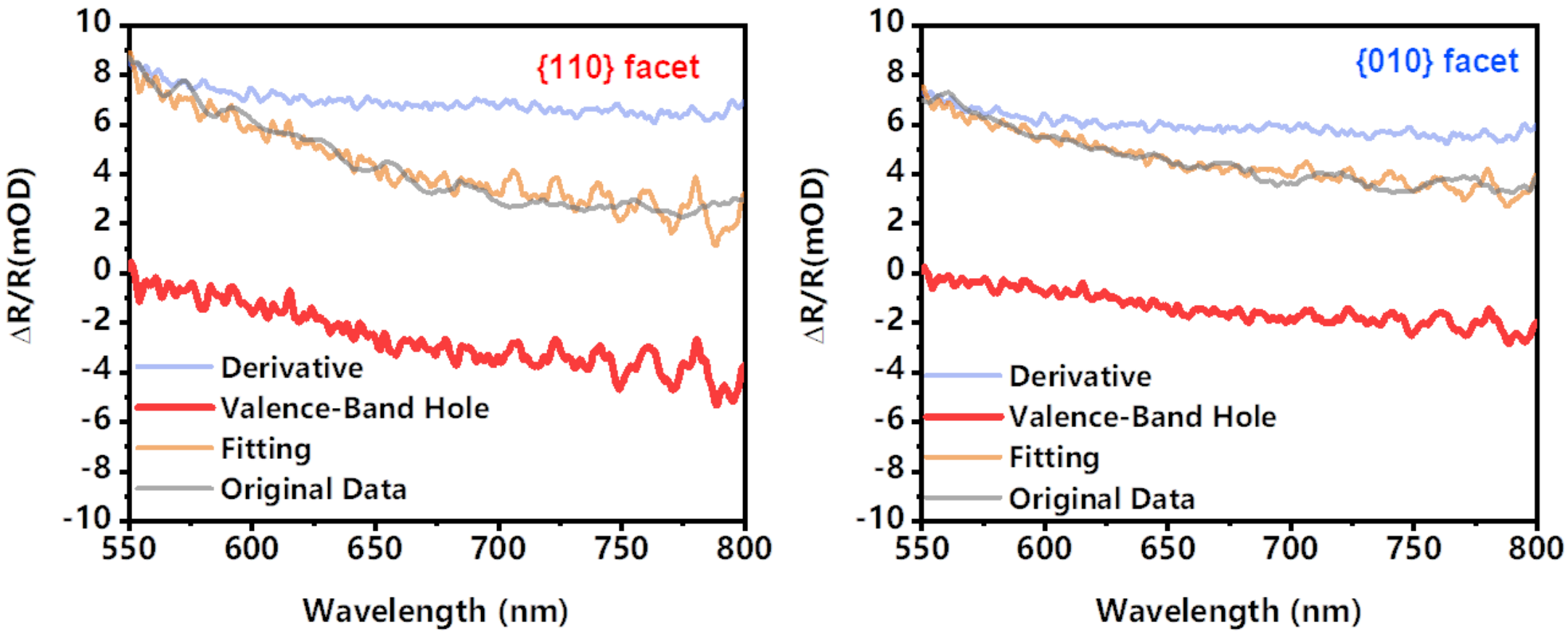

Figure S16. Transient reflection spectra in the below bandgap region and the decomposition results at 50 ps for {110} facet (a) and {010} facets. Blue and red lines represent the tail of the derivative component in the below bandgap region and the valence-band hole signal, respectively. The gray and orange lines are the original spectra and the fitting result of these two components. Valence-band holes preferentially accumulate on {110} facets.

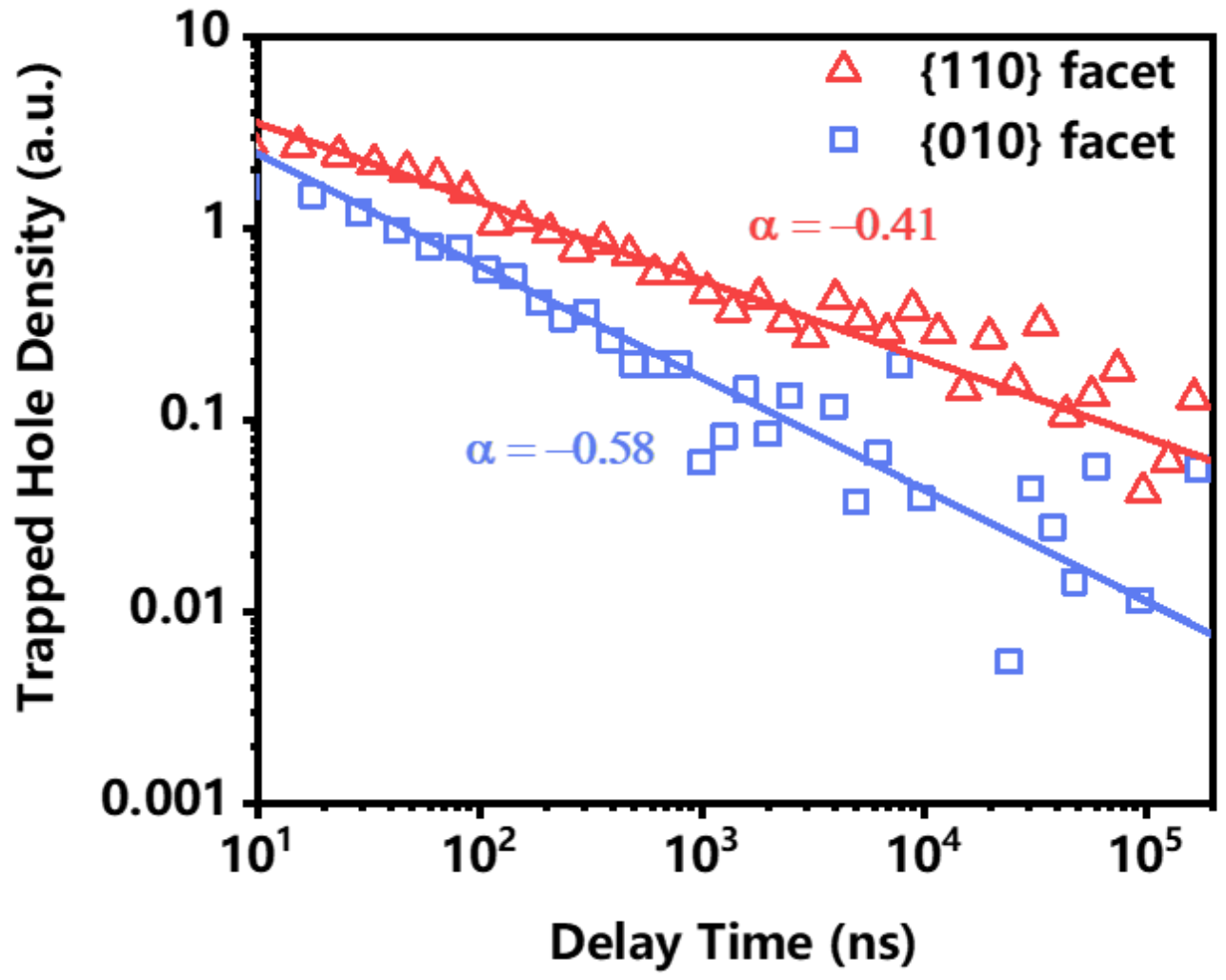

Figure S17. Time evolution of trapped hole signal extracted from transient reflection measurement at {010} and {110} facets in DMF. Both of them decayed in a power law manner at long delay times, which further validates the trapped nature of this signal.

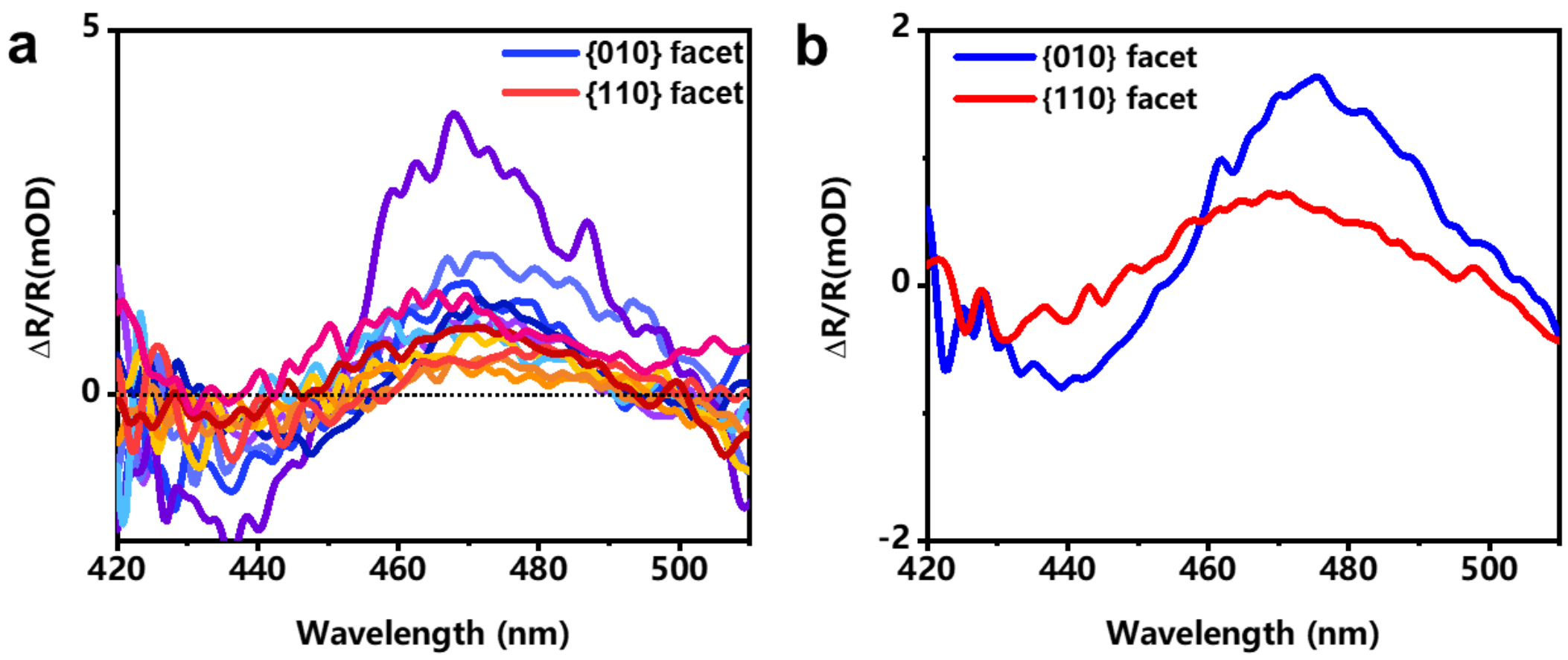

Figure S18. Statistical (**a**) and averaged (**b**) comparison of transient reflection spectra at {010} and {110} facets pumped by 1030 nm femtosecond laser at 100 ps. The transient reflection spectra under sub-

bandgap excitation act as a direct probe of trap state concentration at oxygen lattice sites.

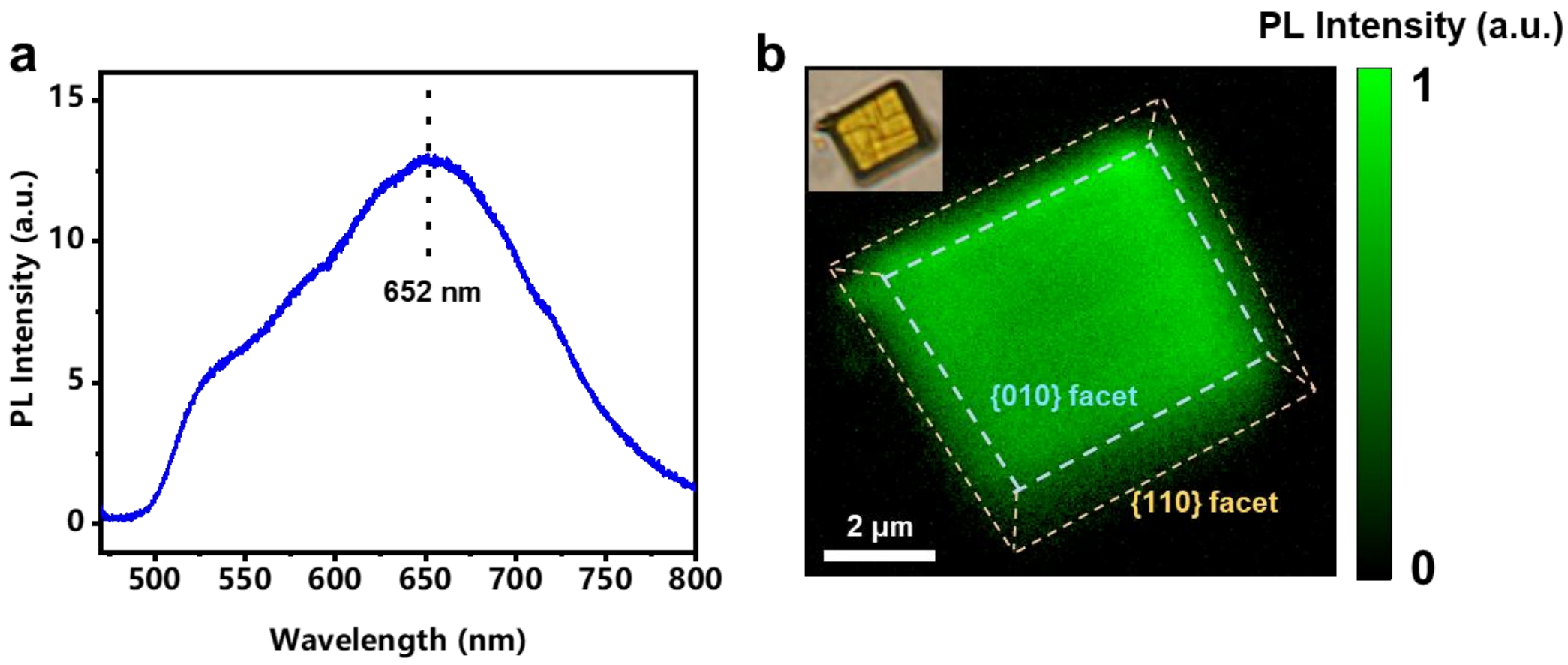

Figure S19. (a) PL emission spectrum of the octahedral $BiVO_4$. (b) PL intensity image of octahedral $BiVO_4$. Scale bar is 2 μm.

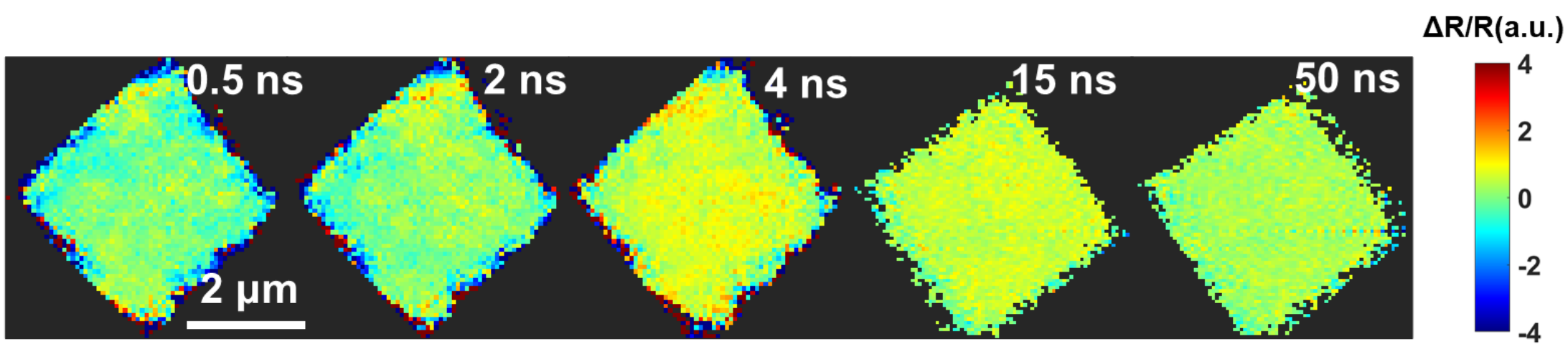

Figure S20. Trapped hole distribution on {010} facets measured in water at different delay times.

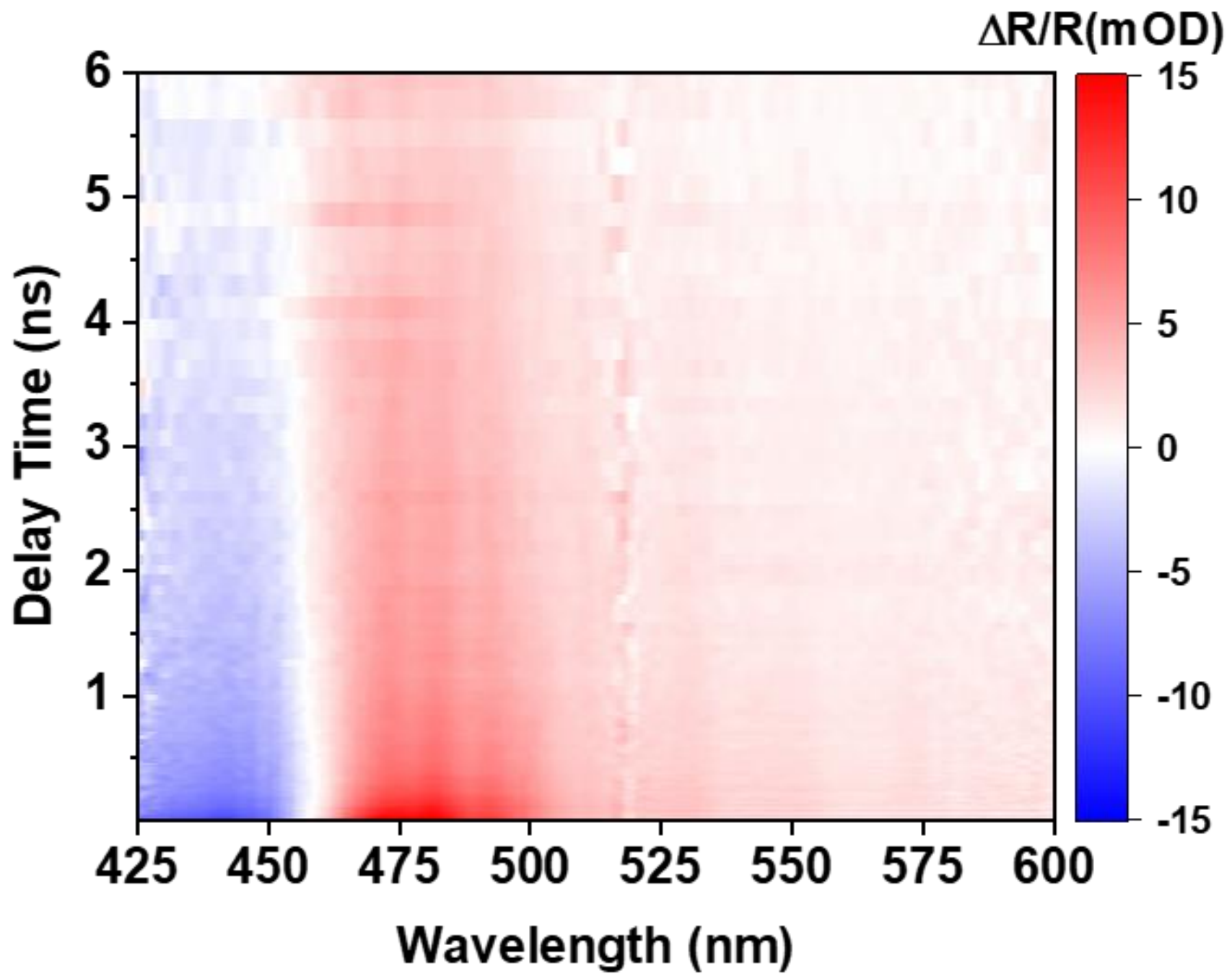

Figure S21. 2D pseudocolor map of transient reflection spectra of decahedral $BiVO_4$ measured on {110} facets in water.

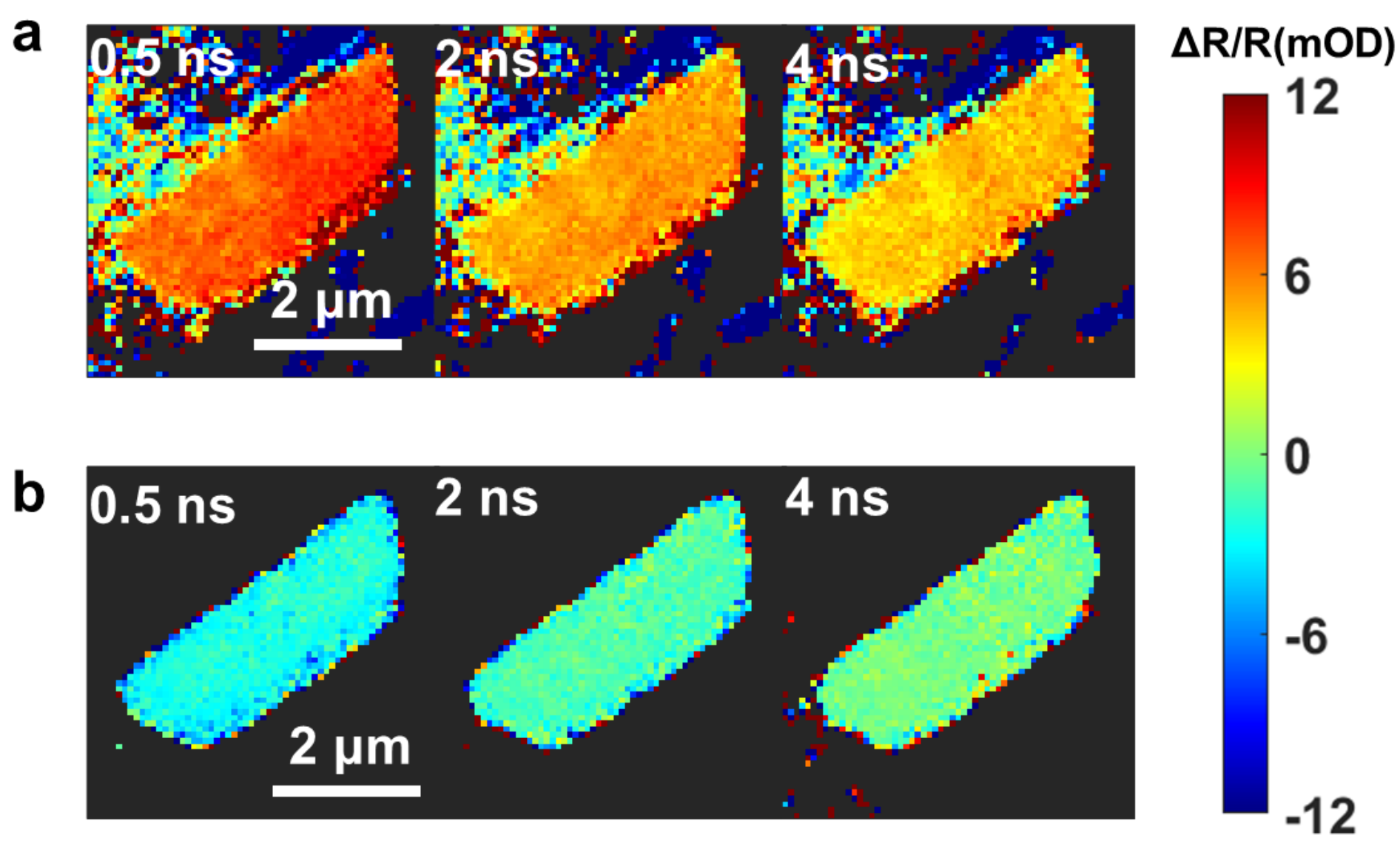

Figure S22. fs-TRM mapping on {110} facets of decahedral $BiVO_4$ in water at 480 nm (**a**) and 430 nm (**b**) at different delay times.

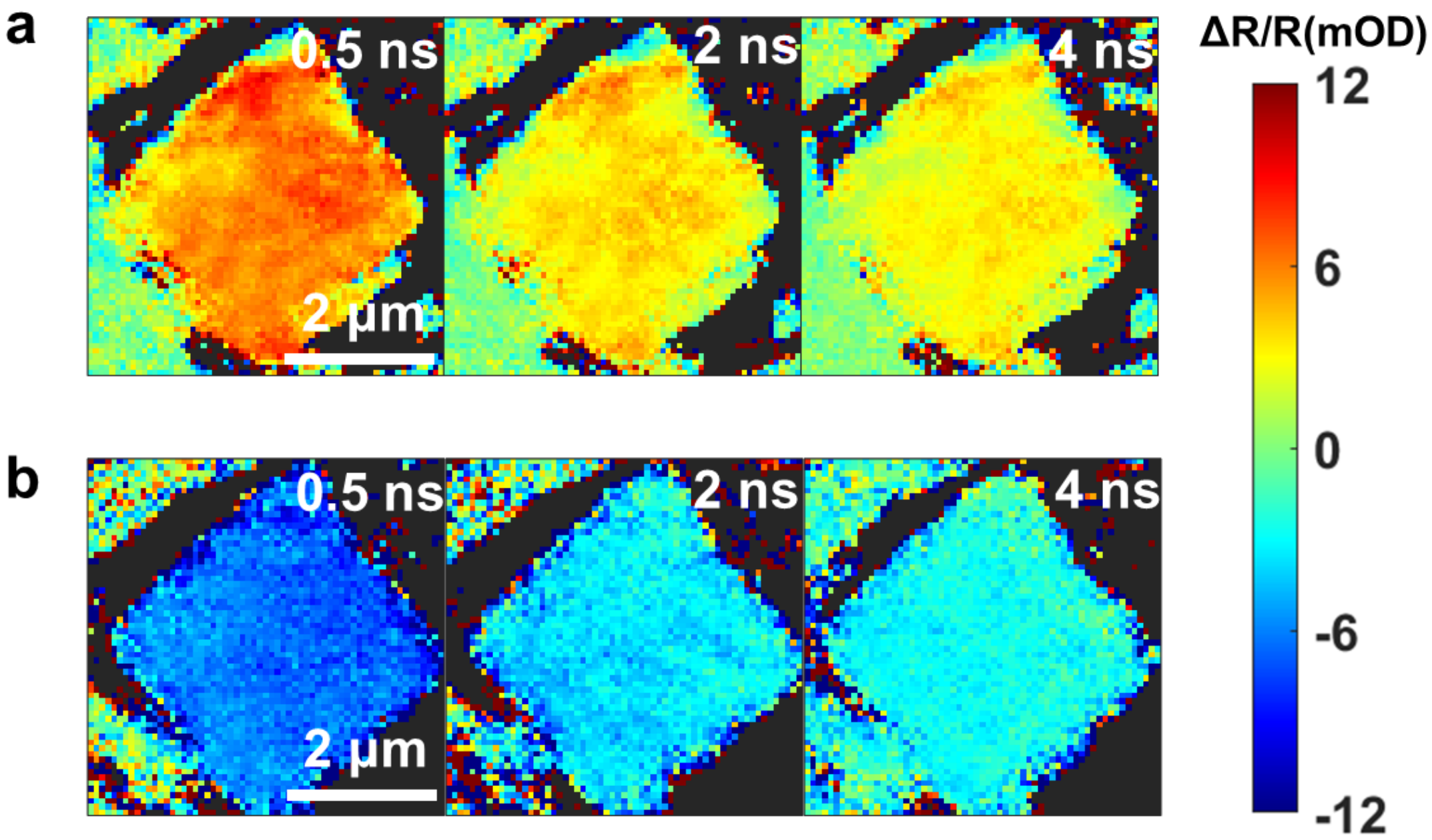

Figure S23. fs-TRM mapping on {010} facets of decahedral $BiVO_4$ in water at 480 nm (**a**) and 430 nm (**b**) at different delay times.

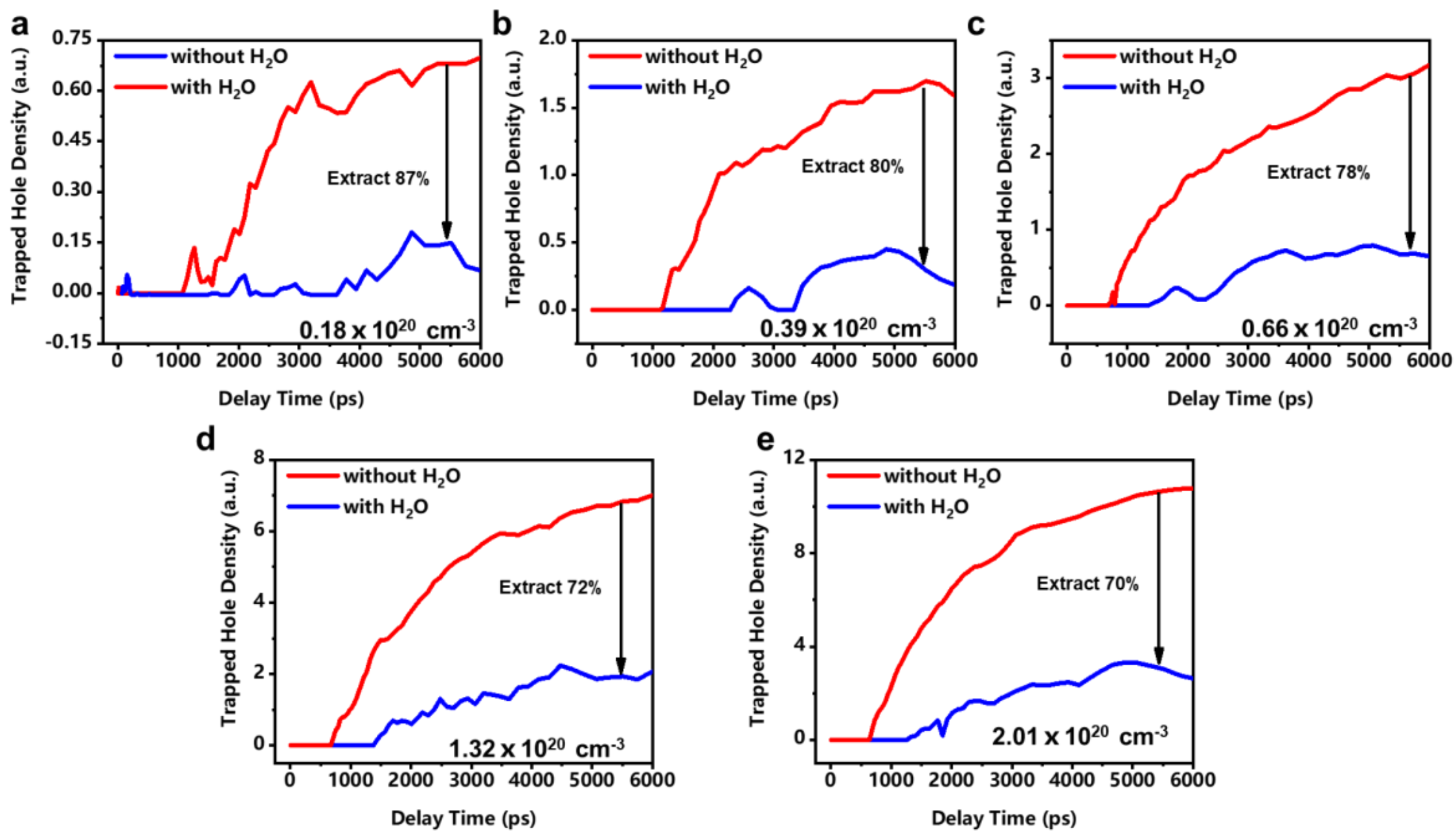

Figure S24. Power Dependence of interfacial hole transfer kinetics at $0.18*10^{20}$ cm^{-3} (**a**), $0.39*10^{20}$ cm^{-3} (**b**), $0.66*10^{20}$ cm^{-3} (**c**), $1.32*10^{20}$ cm^{-3} (**d**) and $2.01*10^{20}$ cm^{-3} (**e**).

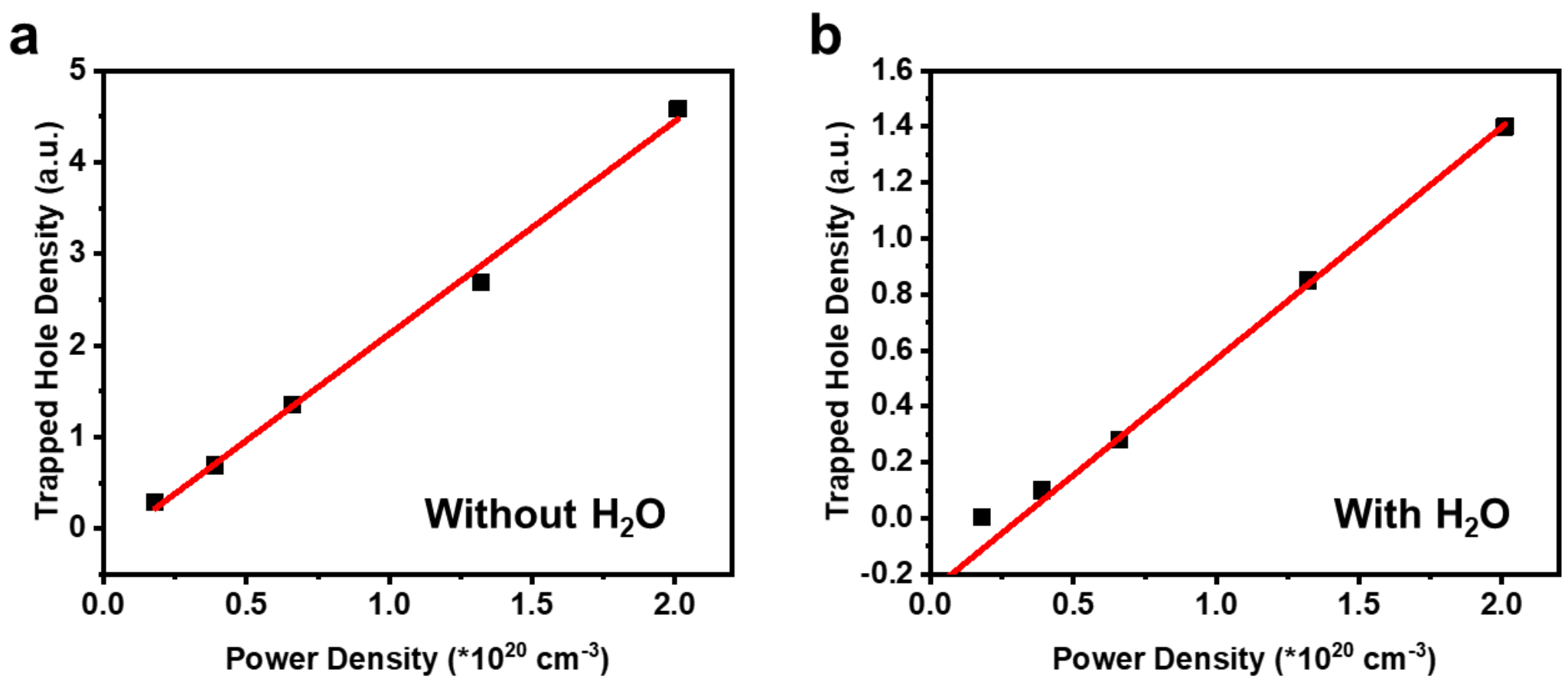

Figure S25. Power Dependence of trapped hole density on {110} facets measured without (**a**) and with (**b**) H_2O. The linearly increased trapped hole density measured without H_2O indicates that trap states are sufficiently abundant for hole trapping. The deviation of trapped hole density from linear trend in H_2O is a signature of efficient and complete interfacial hole extraction under low power density.

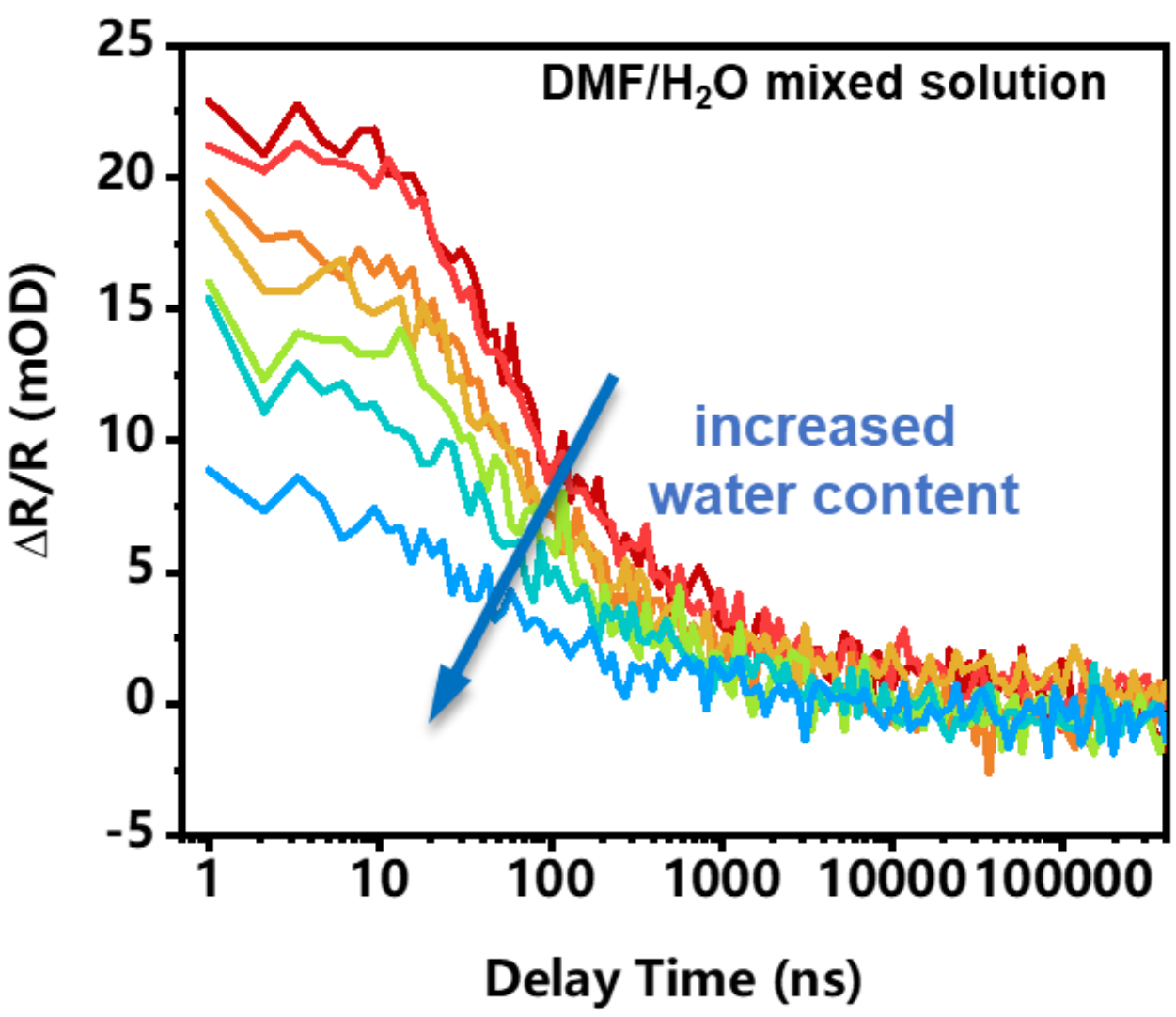

Figure S26. Water content dependence of trapped hole kinetics on {110} facets. A decrease in water content significantly retards interfacial hole extraction.

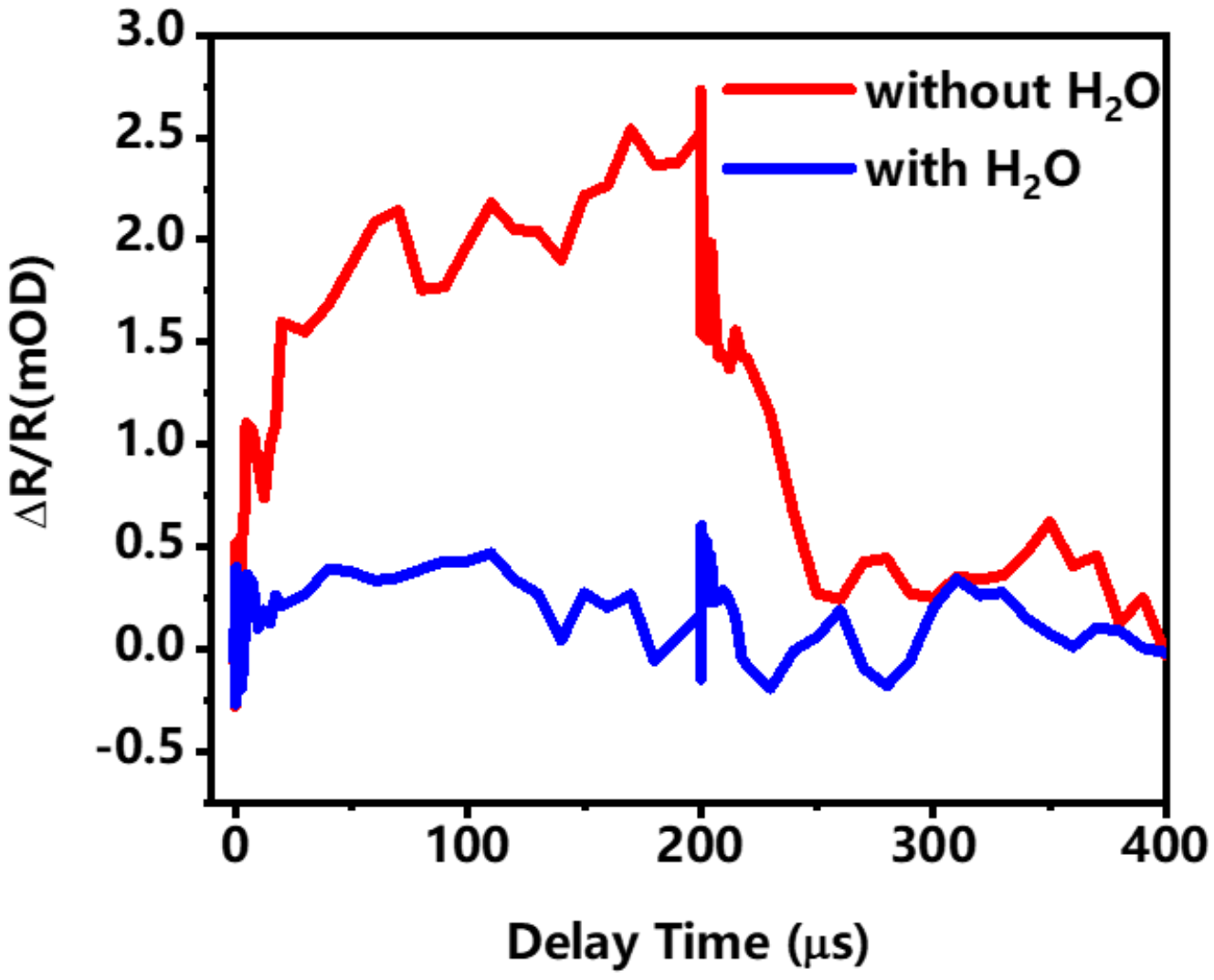

Figure S27. Temporal evolution of trapped hole signal on {110} facets pumped by 450 nm continuous-wave laser under about 100 sun illumination.

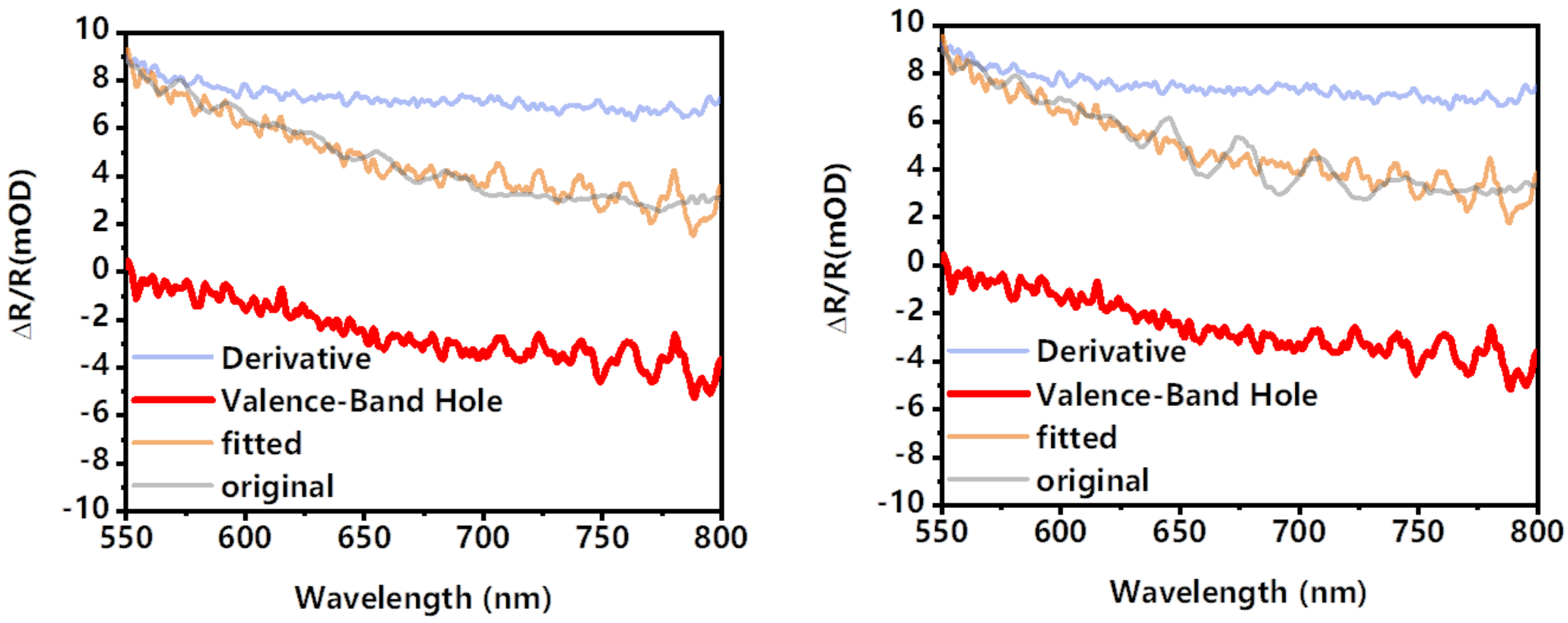

Figure S28. Transient reflection spectra in the below bandgap region and the decomposition results at 200 ps on {110} facets in different solvents. **a,** TR spectra measured in DMF. **b,** TR spectra measured in H_2O. No noticeable difference could be observed between these two spectra, which demonstrates that valence-band holes could not directly transfer to pre-adsorbed H_2O molecules.

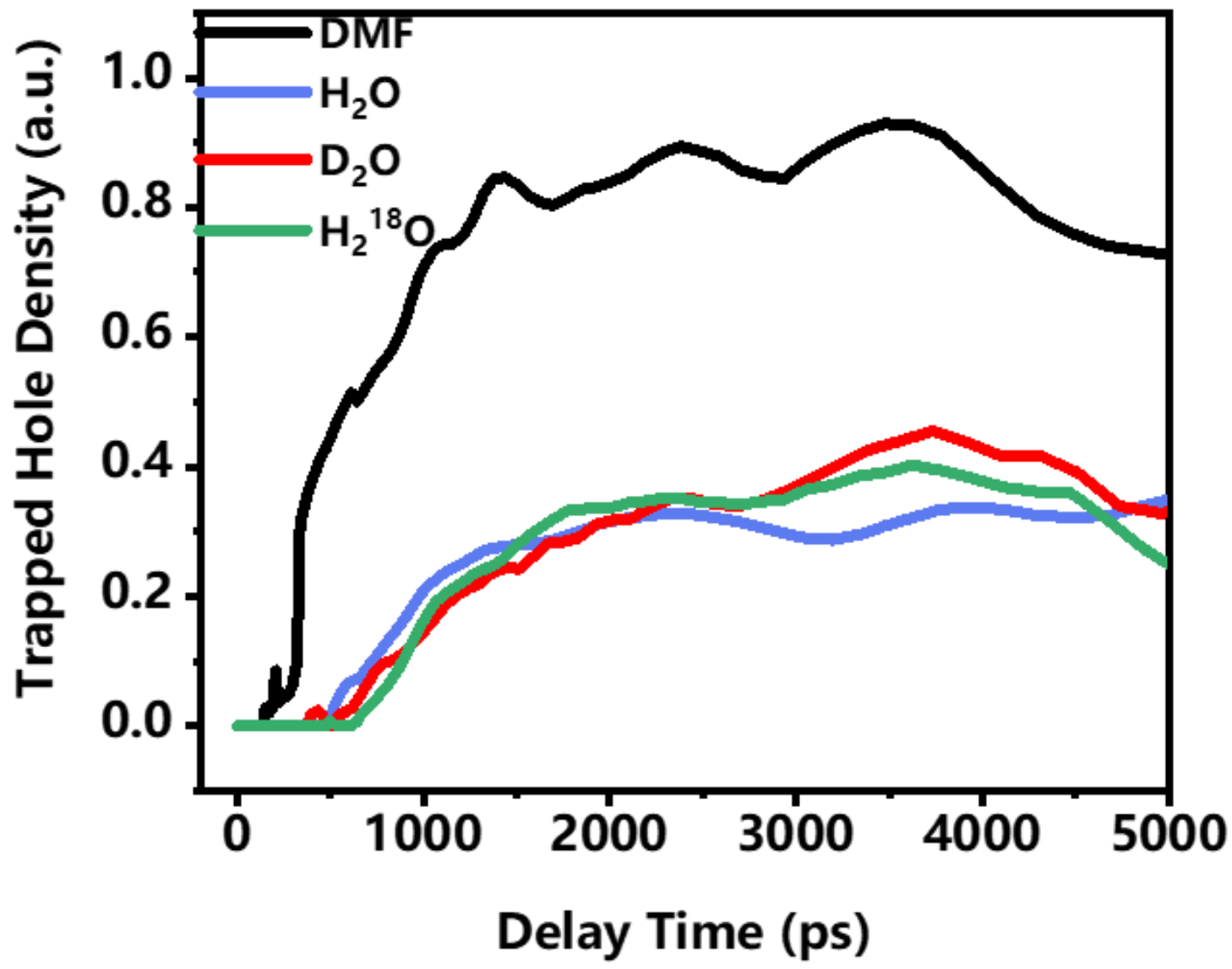

Figure S29. Kinetic isotope effect of the interfacial hole transfer process. No observable change could be detected after isotope substitution.

Table S1 Fitting parameters of interfacial hole transfer in decahedral $BiVO_4$ crystal. k_{tr} and k_r represent the first-order hole trapping rate constant and second-order interfacial hole transfer rate constant respectively. $[H_2O_{ads,0}]$ is the effective initial concentration of pre-adsorbed H_2O.

$k_{tr}(s^{-1})$	$k_r(cm^{-3}s^{-1})$	$[H_2O_{ads,0}](cm^{-3})$
$0.65 * 10^9$	$13.1 * 10^{-10}$	$0.41 * 10^{20}$

Reference:

1. Yan, X.,Chen, C.,Wu, B. et al., Probing the operation of quantum-dot light-emitting diodes using electrically pumped transient absorption spectroscopy. *J Phys Chem Lett* 2024; **15**: 8593-99.
2. Kresse, G.,Joubert, D., From ultrasoft pseudopotentials to the projector augmented-wave method. *Phys Rev B* 1999; **59**: 1758-75.
3. Hafner, J., Ab-initio simulations of materials using VASP: Density-functional theory and beyond. *J Comput Chem* 2008; **29**: 2044-78.
4. Perdew, J. P.,Burke, K.,Ernzerhof, M., Generalized Gradient Approximation made simple. *Phys Rev Lett* 1996; **77**: 3865-68.
5. Grimme, S.,Ehrlich, S.,Goerigk, L., Effect of the damping function in dispersion corrected density functional theory. *J Comput Chem* 2011; **32**: 1456-65.
6. Sun, F.,Deng, Y.,Leng, J. et al., Visualizing ultrafast photogenerated electron and hole separation in facet-engineered bismuth vanadate crystals. *J Am Chem Soc* 2024; **146**: 31106-13.
7. Cooper, J. K.,Reyes-Lillo, S. E.,Hess, L. H. et al., Physical origins of the transient absorption spectra and dynamics in thin-film semiconductors: The case of $BiVO_4$. *J Phys Chem C* 2018; **122**: 20642-52.
8. Kunzelmann, V. F.,Jiang, C.-M.,Ihrke, I. et al., Solution-based synthesis of wafer-scale epitaxial $BiVO_4$ thin films exhibiting high structural and optoelectronic quality. *J Mater Chem A* 2022; **10**: 12026-34.
9. Zhao, Z.,Li, Z.,Zou, Z., Electronic structure and optical properties of monoclinic clinobisvanite $BiVO_4$. *Phys Chem Chem Phys* 2011; **13**: 4746–53.
10. Das, T.,Rocquefelte, X.,Laskowski, R. et al., Investigation of the optical and excitonic properties of the visible light-driven photocatalytic $BiVO_4$ material. *Chem Mater* 2017; **29**: 3380-86.
11. Chaudhuri, A.,Mandal, L.,Chi, X. et al., Direct observation of anisotropic small-hole polarons in an orthorhombic structure of $BiVO_4$ films. *Phys Rev B* 2018; **97**: 195150
